# The Arabidopsis apyrase AtAPY1 is localized in the Golgi instead of the extracellular space

**DOI:** 10.1186/1471-2229-12-123

**Published:** 2012-07-31

**Authors:** Madlen Schiller, Carolin Massalski, Thomas Kurth, Iris Steinebrunner

**Affiliations:** 1 Department of Biology, Section of Molecular Biotechnology, Technische Universität Dresden, Helmholtzstraße 10, Dresden 01069, Germany; 2 DFG-Center for Regenerative Therapies Dresden (CRTD), Technische Universität Dresden, Fetscherstraße 105, Dresden 01307, Germany

**Keywords:** Apyrase, Regulation of growth, Golgi, Extracellular ATP, Transmembrane protein, SNAP-tag, GFP-tag, Co-localization, Substrate specificity

## Abstract

**Background:**

The two highly similar Arabidopsis apyrases AtAPY1 and AtAPY2 were previously shown to be involved in plant growth and development, evidently by regulating extracellular ATP signals. The subcellular localization of AtAPY1 was investigated to corroborate an extracellular function.

**Results:**

Transgenic Arabidopsis lines expressing *AtAPY1* fused to the SNAP-(O^6^-alkylguanine-DNA alkyltransferase)-tag were used for indirect immunofluorescence and AtAPY1 was detected in punctate structures within the cell. The same signal pattern was found in seedlings stably overexpressing *AtAPY1-GFP* by indirect immunofluorescence and live imaging. In order to identify the nature of the AtAPY1-positive structures, *AtAPY1-GFP* expressing seedlings were treated with the endocytic marker stain FM4-64 (N-(3-triethylammoniumpropyl)-4-(p-diethylaminophenyl-hexatrienyl)-pyridinium dibromide) and crossed with a transgenic line expressing the *trans*-Golgi marker *Rab E1d*. Neither FM4-64 nor Rab E1d co-localized with AtAPY1. However, live imaging of transgenic Arabidopsis lines expressing *AtAPY1-GFP* and either the fluorescent protein-tagged Golgi marker *Membrin 12*, *Syntaxin of plants 32* or *Golgi transport 1 protein homolog* showed co-localization. The Golgi localization was confirmed by immunogold labeling of AtAPY1-GFP. There was no indication of extracellular AtAPY1 by indirect immunofluorescence using antibodies against SNAP and GFP, live imaging of AtAPY1-GFP and immunogold labeling of AtAPY1-GFP. Activity assays with AtAPY1-GFP revealed GDP, UDP and IDP as substrates, but neither ATP nor ADP. To determine if AtAPY1 is a soluble or membrane protein, microsomal membranes were isolated and treated with various solubilizing agents. Only SDS and urea (not alkaline or high salt conditions) were able to release the AtAPY1 protein from microsomal membranes.

**Conclusions:**

AtAPY1 is an integral Golgi protein with the substrate specificity typical for Golgi apyrases. It is therefore not likely to regulate extracellular nucleotide signals as previously thought. We propose instead that AtAPY1 exerts its growth and developmental effects by possibly regulating glycosylation reactions in the Golgi.

## Background

The term “apyrase” (adenosine pyrophosphatase) for an enzyme cleaving the phosphoanhydride bonds of ATP and ADP was coined by Otto Meyerhof in 1945 [1]. Decades later, the alternative name “NTPDase” (nucleoside triphosphate diphosphohydrolase) was officially proposed [2] because apyrases hydrolyze a wide range of nucleoside tri- and diphosphates (reviewed in [3]). Apyrases have been found in many pro- and eukaryotes (reviewed in [3]), and they all share highly conserved regions [4]. In plants, the postulated functions are diverse and include nodulation [5-9], resistance to xenobiotics [10], phosphate scavenging [11] and growth [12-16]. Each eukaryotic genome screened for the presence of apyrase genes holds at least two candidates. In *Arabidopsis thaliana*, a total of seven apyrase gene candidates exist. Our research focused on the function of the two Arabidopsis apyrase genes *AtAPY1* and *AtAPY2*, whose corresponding proteins share an identity of 87% amino acids. Knocking out one of the two apyrase genes by T-DNA (transfer DNA) insertion resulting in an *apy1* or *apy2* single knockout (SKO) caused no obvious differences in phenotype compared with the wild type (WT) [17], but knocking out both *AtAPY1* and *AtAPY2* inhibited pollen germination [17] and was seedling-lethal [18]. Overexpression of either *AtAPY1* or *AtAPY2* led to more vigorous growth of hypocotyls and pollen tubes [12]. Suppression of expression, however, by RNA interference targeting *AtAPY1* in the *apy2* SKO background, inhibited growth throughout the whole plant and especially in the hypocotyls and roots [12]. Several lines of evidence suggested that these growth effects are mediated by AtAPY1 and AtAPY2 regulating extracellular ATP (eATP) signals [12]: Apyrase activity, measured in the extracellular matrix (ECM) of growing pollen tubes, could be reduced by adding chemical inhibitors or polyclonal antibodies directed against AtAPY1. The reduction in activity simultaneously raised eATP levels and reduced pollen tube growth [12]. These findings explained the inhibition of growth when the expression of *AtAPY1* and *AtAPY2* is suppressed or shut off and provided the first direct evidence that apyrases function as regulators of extracellular nucleotides such as eATP in plants. In the animal field, the direct link between ecto-apyrases and [eATP] had already been shown [19]. Similarly, eATP was already known to serve as signaling molecule in animals (reviewed in [20]) before it became recognized as such in plants in the past decade (reviewed in [21-23]).

The objective of this study was to confirm the extracellular function of the two Arabidopsis apyrases AtAPY1 and AtAPY2 by their localization to the plasma membrane or the apoplast. Since *AtAPY1* and *AtAPY2* were shown to be functionally redundant in their ability to rescue pollen germination of double knockout apyrase (DKO) pollen [17] and seedling viability in DKO mutants [18], an overlapping subcellular localization of the two apyrases was likely. Therefore, this study focused on the localization of only one apyrase.

Stable Arabidopsis lines were generated expressing *AtAPY1* fused to either one of two tag sequences, *SNAP* or *GFP*. For the identification of the AtAPY1-positive compartments, organelle-specific marker proteins were co-expressed and immunogold labeling was used. Unexpectedly, the apyrase was not localized to the plasma membrane or cell wall, but to the Golgi apparatus.

## Methods

### Plant material and growth conditions

For all experiments, the *A. thaliana* ecotype Wassilewskija was used as the WT control. Seedlings were grown for one week under sterile conditions on agar plates (4.3 gL^-1^Murashige Skoog (MS) salts, 0.5 g L^-1^ MES, pH 5.7 (adjusted with KOH), 1% (w/v) sucrose, 0.8% (w/v) agar) or in liquid medium (see above, without agar) under shaking (80 rpm). After one week on agar plates, seedlings were transferred to soil (Einheitserde, type P, Pätzer Inc., Sinntal-Jossa, Germany) and grown at 24°C and a 16-h photoperiod at 100 μmol photons m^- 2^ s^-1^.

### Genotypic background and terminology of apyrase mutants

The term SKO refers to the homozygous presence of the null alleles of either the *AtAPY1* gene (= *apy1/apy1*) or the *AtAPY2* gene (= *apy2/apy2*). The T-DNA null mutations *apy1* and *apy2* refer to the mutant alleles *apy1-1* and *apy2-1*, respectively, as described in Steinebrunner et al. [17]. The symbol “+” refers to the WT counterpart of the mutant allele. Two types of apyrase DKO mutants were generated: DKO-SNAP (= *apy1/apy1; apy2/apy2*; *SPIK::AtAPY2*; *AtAPY1::AtAPY1-SNAP*) and DKO-GFP (= *apy1/apy1; apy2/apy2*; *SPIK::AtAPY2*; *35S::AtAPY1-GFP*). Both types of DKO mutants carried the *AtAPY2* gene under the control of the *SPIK* promoter. The details of the construct *SPIK::AtAPY2* were published in [18]. *SPIK* is the promoter region of the shaker pollen inward K^+^ channel gene expressed specifically in pollen and pollen tubes [24].

### Generation of *AtAPY1-SNAP*-complemented apyrase DKO mutants (= DKO-SNAP)

The open reading frame (ORF) of *AtAPY1* fused to the SNAP-tag sequence was cloned under the control of the native *AtAPY1* promoter region (nt −10 to −1959; with −1 corresponding to the first nucleotide upstream of the adenine of the *AtAPY1* start codon) with the Gateway technology (Invitrogen). The *AtAPY1-SNAP* sequence is shown in the Additional file 1. For the SNAP-tagging, the stop codon was removed from the *AtAPY1* sequence to create a C-terminal fusion to the tag. The vector pSNAP-tag(m) (New England Biolabs) was used as the PCR template for the *SNAP*-tag sequence. The primer pair for the amplification produced a 531-bp product and contained the following *SNAP*-specific sequences: 5’-GACTGCGAAATGAAGCGCA-3’ (SNAPlokattB3F; forward) and 5’-TTAAGGCTTGCCCAGTCTGTG-3’ (SNAPlokattB2R; reverse). The reverse primer introduced the stop codon. The entry clone for each of the three DNA elements was generated by recombining the respective PCR product with the matching pDONR vector (Invitrogen). The necessary recombination sites were introduced into the PCR product through the primer sequences. The three entry clones were recombined with the binary destination vector pGWB501 [25] to form the final construct *AtAPY1::AtAPY1-SNAP*. The sequences of the entry clones and the expression clone were confirmed by sequencing. The *Agrobacterium tumefaciens* strain GV3101 [26] was transformed with the expression clone and then used for transformation of apyrase mutants which were hemizygous for the *apy1* mutation (= *+/apy1*), homozygous for the *apy2* mutation (= *apy2/apy2*; SKO) and contained the construct *SPIK::AtAPY2*. The plants were transformed by the floral dip method [27]. Transgenic lines (T1 generation) were grown on agar plates containing hygromycin (50 μg mL^-1^), phosphinothricin (PPT) (10 μg mL^-1^) and kanamycin (30 μg mL^-1^). Hygromycin selected for the presence of *AtAPY1::AtAPY1-SNAP*, PPT for the presence of *SPIK::AtAPY2* and kanamycin for the presence of *apy1* or *apy2.*

### Generation of *AtAPY1-GFP*-complemented apyrase DKO mutants (= DKO-GFP)

The cloning of the *35S::AtAPY1-GFP* construct is described in detail in [28]. The ORF of *AtAPY1* without the stop codon was amplified by PCR and cloned into the pQE-30 vector (Qiagen). For the amplification of the *GFP* cDNA, the pBIN mGFP5-ER [29] served as the template. The PCR product was cloned in frame with the *AtAPY1* sequence already present in pQE-30 to enable a translational fusion of GFP with the C-terminus of AtAPY1. The resulting conjugated *AtAPY1-GFP* ORF was amplified and subcloned into the TOPO pCR2.1 vector (Invitrogen). The *AtAPY1-GFP* cDNA was released by *Eco*RI digestion and cloned into the binary vector pLBJ21 [30]. The *AtAPY1-GFP* sequence is available as Additional file 2. The transgenic line expressing the *GFP*-tag alone is available from the Arabidopsis Resource Center (stock number CS9114).

WT Wassilewskija plants were transformed with the help of agrobacteria containing the recombinant construct by the floral dip method [27]. Transformants (T1 generation) were selected on agar plates containing kanamycin (30 μg mL^-1^). For genetic complementation experiments, homozygous *AtAPY1-GFP* transgenic lines were crossed with *apy1* SKO plants hemizygous for the *apy2* mutation and carrying the *AtAPY2* cDNA under the control of the *SPIK* promoter (= *apy1/apy1; +/apy2*; *SPIK::AtAPY2*). Kanamycin-resistant progeny were genotyped by PCR. *Apy1* SKO plants containing the *AtAPY1-GFP* construct (= *apy1/apy1; +/+; 35S::AtAPY1-GFP*) were crossed with *apy2* SKO plants hemizygous for the *apy1* mutation and expressing *AtAPY2* under the control of the *SPIK* promoter (= *+/apy1; apy2/apy2; SPIK::AtAPY2*). The progeny were selected on kanamycin and PPT.

### Screening for complemented apyrase DKO mutants

For the screening for complemented apyrase DKO-SNAP and DKO-GFP mutants, genomic DNA was extracted from candidate plants as described elsewhere [31]. For detection of *AtAPY1*, *AtAPY2*, *apy1, apy2* and *SPIK::AtAPY2*, the used primer combinations are described in [18], except for the reverse *AtAPY1*-specific primer which was changed to A1I1R (5’-GCGAGCTAGAAATACCACC-3’) yielding a PCR product of 1 kb. The presence of the *AtAPY1::AtAPY1-SNAP* construct was confirmed using the *SNAP*-specific primer SNAPlokattB2R and the *AtAPY1*-specific primer A1E9F (5’-CCACTAGGAAGCGCAATAGA-3’) located in exon 9. The *35S::AtAPY1-GFP* construct was amplified using the *AtAPY1* forward primer A1E9F and a reverse primer located in the *GFP* sequence (GFP_rev 5’-TGTATAGTTCATCCATGCCATG-3’) resulting in a PCR product of 0.7 kb.

### Generation of transgenic lines co-expressing *AtAPY1-GFP* and either *YFP-(yellow fluorescent)-Rab E1d, -SYP32, -Got1p homolog* or *RFP - (red fluorescent protein)-MEMB12*

Four transgenic lines generated by Geldner et al. [32] expressing either the marker *YFP-Rab E1d*, *YFP-Got1phomolog*, *YFP-SYP32* or *RFP-MEMB12* under the control of the *UBQ10* promoter were obtained from the Nottingham Arabidopsis Stock Centre. The lines were validated by antibiotic selection and genomic PCR as suggested by Geldner et al. [32]. Homozygous transgenic *35S::AtAPY1-GFP* plants were crossed with either homozygous *UBQ10::YFP-Got1p homolog, YFP-SYP32* or *RFP-MEMB12* plants. The F1 progeny were selected on agar plates containing kanamycin (30 μg mL^-1^) and either PPT (10 μg mL^-1^) for the crosses with the *YFP* fusion constructs or hygromycin (50 μg mL^-1^) for the crosses with the *RFP* fusion construct. Double resistant F1 seedlings were genotyped for the existence of the desired fusion constructs before analyzing them by confocal microscopy.

### Protoplast preparation

Ten-day-old *35S::AtAPY1-GFP* transgenic seedlings from a liquid culture were digested over night at 18°C in buffer (0.4 M sorbitol, 20 mM HEPES-KOH pH 7.6, 2.5 mM EDTA, 5 mM MgCl_2_, 10 mM NaHCO_3_, 0.1% (w/v) bovine serum albumin, freshly added 1.6% (w/v) cellulase Onozuka RS (Duchefa) and 1.6% (w/v) macerozyme R-10 from *Rhizopus sp*. (Serva)) [33].

### Whole mount immunofluorescence of AtAPY1-SNAP

Ten-day-old DKO-SNAP seedlings were fixed in 4% (w/v) paraformaldehyde (PFA) for 1 h and treated with 1% (w/v) cellulase Onozuka RS (Duchefa) and 1% (w/v) macerozyme R-10 from *Rhizopus sp*. (Serva) for 15 min at 37°C. After washing twice with phosphate buffered saline (PBS), the samples were treated with 1% (v/v) Triton X-100 for 1 h at room temperature (RT). To inactivate all endogenous peroxidases, the samples were incubated in methanol:hydrogen peroxide (200:1, v/v) for 30 min at RT in the dark. After washing with water, the seedlings were treated with 96% (v/v) ethanol for 1 min, washed twice with PBS and blocked with 1% (w/v) skim milk (Fluka) for 30 min. Incubation with polyclonal rabbit α-SNAP antibody (Open Biosystems, Huntsville, Alabama, USA) (1:50 in 1% (w/v) skim milk) followed for at least 1 h at RT. After washing three times with PBS, the seedlings were incubated for at least 1 h at RT with goat α-rabbit-IgG conjugated with horseradish peroxidase (HRP) (GE Healthcare) diluted 1:800 in 1% (w/v) skim milk. After removing the secondary antibody by washing three times with PBS, the seedlings were treated with 2 μL fluorescein isothiocyanate (FITC) tyramides (1 mg mL^-1^) in 50 mL amplification buffer [34] for at least 1 h at RT or at 4°C overnight in the dark. FITC tyramides were prepared according to Pernthaler et al. [34] and kindly provided by Kerstin Röske. The samples were washed three times with PBS and stored for imaging in PBS.

### Whole mount immunofluorescence of AtAPY1-GFP

Root tips of six-day-old *35S::AtAPY1-GFP* transgenic seedlings were fixed with 4% (w/v) PFA in 0.1 M phosphate buffer (PB, pH 7.4) for 30 min, 2 h in 8% (w/v) PFA ( [35]; Y-D Stierhof, personal communication) followed by postfixation in 80% methanol/20% DMSO (Dent’s fixative, [36]). Fixed samples were whole-mount immunolabeled with rabbit α-GFP (TP 401; Lot no. 071519 from Torrey Pines, 1:100) and goat α-rabbit Alexa Fluor 488 (Invitrogen; Lot no. 430222; 1:100) and then embedded in Technovit 7100 as previously described [37]. Three-μm sections were analyzed with the fluorescence microscope Keyence BZ 8000 (Additional file 3).

### Treatment with FM4-64 and alkaline pH

Seven- to ten-day-old *35S::AtAPY1-GFP* transgenic seedlings were vacuum-infiltrated with 15 μM FM4-64 (Invitrogen) in 1 M Tris–HCl pH 8.0. For the alkali treatment, two approaches were taken: (1) *35S::AtAPY1-GFP* seedlings were cultured in regular liquid MS medium (pH 5.7) for five days, transferred to alkaline MS medium (pH 8.1) and grown for three more days before imaging [38] or (2) the seedlings were grown in regular liquid MS medium (pH 5.7) for eight days and infiltrated with tap water or Tris buffered saline (TBS) pH 7.5 for at least 2 h before imaging.

### CLSM (confocal laser scanning microscopy)

For the imaging of DKO-SNAP plants (10 d old), the Leica confocal and multiphoton microscope TCS SP5 MP was used. To avoid bleaching of FITC, the samples were mounted with antifade (0.233 g 1,4-diazabicyclo(2.2.2)octane (Sigma) in 200 μL 1 M Tris–HCl pH 8.0, 800 μL water, 9 mL glycerine) [39]. The FITC signals were captured using a Leica water immersion objective (HCX PL APO 63x/1.2 Water Lbd.Bl.). The detector range was set to 494 to 530 nm. The autofluorescence of the WT seedlings (10 d old) was captured with the same parameters and settings as described for FITC. Spectral images of the WT and the DKO-SNAP samples were analyzed by linear unmixing with the dye separation tool of the Leica software (LAS1.8.2) to identify FITC-specific signals.

Transgenic plant material containing the *35S::AtAPY1-GFP* construct were imaged with the Zeiss Axio Imager connected to the laser scanning microscope LSM 710 or 780 (Carl Zeiss). Six- to 10-d-old seedlings were imaged in water and protoplasts in sorbitol buffer pH 7.6 (see Protoplast preparation) without the enzymes. The images were analyzed with the Zeiss Zen 2009 and Fiji [40] software. Zeiss water immersion objectives (C-Apochromat 40x/1.20 W Korr M27 or C-Apochromat 63x/1.20 W Korr M27) were used. Chlorophyll fluorescence was detected between 601 to 708 nm after excitation with the 594-nm excitation line of a helium-neon (HeNe) laser. GFP was excited with the 488 nm argon laser multiline and the emission was collected between 490 and 520 nm. YFP was excited with the 514-nm excitation line of an argon laser (multi-beam splitter 514/561) and the emission was collected between 535 and 580 nm. RFP was excited at 561 nm and its fluorescence detected in the 570 to 630-nm range. For co-imaging of GFP with either YFP or RFP, the line sequential imaging mode was chosen with rapid switching between the two exciting laser lines. For detection of GFP and YFP in one sample, GFP was excited at 458 nm and YFP as described above (multi-beam splitter 458/514/594). The fluorescence of GFP and FM4-64 was imaged simultaneously by using the exciting laser line 488 nm, but separate emission detectors (490 – 543 nm and 667 – 746 nm, respectively). For all dual labelings, narrow detector entrance slit bandwidths were chosen to avoid bleed-through of fluorescence emissions. Bright field-type images were acquired with the transmitted light detector.

### Immunogold labeling of AtAPY1-GFP

Root tips of six-day-old *35S::AtAPY1-GFP* transgenic seedlings were fixed as described under “Whole mount immunofluorescence of AtAPY1-GFP”. Fixed samples were processed for Tokuyasu cryo-sectioning as described [41]. In brief, root tips were washed several times in PB, infiltrated stepwise into gelatine and cooled down on ice. Blocks with single root tips were cut on ice, incubated in 2.3 M sucrose in water for 24 h at 4°C, mounted on Pins (Leica no. 16701950) and plunge-frozen in liquid nitrogen. One-hundred-nm-thin sections were cut on a Leica UC6 equipped with a FC6 cryo-chamber and picked up in methyl cellulose sucrose (1 part 2% (w/v) methyl cellulose (Sigma M-6385, 25 centipoises) + 1 part 2.3 M sucrose). For immunogold labeling, the grids were placed upside down on drops of PBS in a 37°C incubator for 20 min, washed with 0.1% (w/v) glycine in PBS (5x1min), blocked with 1%(w/v) bovine serum albumin in PBS (2x5min) and incubated with primary antibodies for 1 h (α-GFP: TP 401 from Torrey Pines, 1:50, or ab290 from Abcam, 1:50). After washes in PBS (4x2min), the sections were incubated with Protein A conjugated to 10-nm or 6-nm gold for 1 h, washed again in PBS (3x5s, 4x2min) and postfixed in 1% (v/v) glutaraldehyde (5 min). The sections were washed with distilled water (10x1min), stained with neutral uranyl oxalate (2% (w/v) UA in 0.15 M oxalic acid, pH 7.0) for 5 min, washed briefly in water and incubated in methyl cellulose uranyl acetate (9 parts 2% (w/v) MC + 1 part 4% (w/v) UA, pH 4) on ice for 5 min. Finally, grids were looped out, the MC/UA film was reduced to an even thin film and air-dried. Sections were analyzed on a Philips Morgagni 268 (FEI) at 80 kV and images were taken with the MegaView III digital camera (Olympus). Areas were calculated using the ITEM-software (Olympus). Alternatively, 200-nm-thin sections were mounted on glass slides and stained with α-GFP and goat α-rabbit Alexa Fluor 488 for fluorescence analysis on a Keyence BZ 8000 fluorescence microscope.

### Co-localization analysis

Transgenic plants co-expressing *AtAPY1-GFP* and either *RFP-MEMB12*, *YFP-SYP32*, *YFP-GOT1p homolog* or *YFP-Rab E1d* were imaged by confocal microscopy and the obtained dual-channel images analyzed with ImageJ [40]. The corresponding scatterplots and Pearson’s correlation coefficients were generated with the “Colocalization Threshold” and “Coloc2” tool of ImageJ.

### Purification of AtAPY1-GFP

For cultivation of starting material, 50 mg of seeds were grown in 50 mL of liquid medium in a 250-mL flask for 10 to 12 d. Seedlings were ground to a fine powder in liquid nitrogen using a mortar and pestle. For each gram of plant material, 250 to 375 μL ice-cold Tris-MES buffer (10 mM Tris, 2 mM MgCl_2_, 30 mM KCl, pH 6.5, adjusted with 1 mM MES pH 3) were added. The cell homogenate was allowed to thaw at RT and filtered through a fine mesh (Miracloth, Calbiochem). The filtrate was subjected to centrifugation at 1,000 *g* and 4°C for 10 min to remove debris. The supernatant was centrifuged at 8,000 *g* and 4°C for 10 min and the pellet was discarded. The supernatant was mixed 1:1 with 100% (v/v) glycerol to retain enzymatic activity and stored at −80°C. For purification of AtAPY1-GFP, 96-well microtiter plates coated with α-GFP antibodies (GFP-multiTrap plates, ChromoTek, Planegg-Martinsried, Germany) were used. Two hundred microliters of protein extract (4–6 μg μL^-1^) were added per well and incubated at 4°C under shaking (500 rpm) for 2 h or overnight. Unbound proteins were removed by washing the wells three times with 300 μL of ice-cold Tris-MES buffer.

### Apyrase activity assay

To determine the apyrase activity, an assay based on Tognoli et al. [42] was used. The nucleotide substrates were purchased from Sigma and the stock solutions were prepared in water. The nucleotides were diluted in Tris-MES buffer (pH 6.5 or pH 5.5) to the desired concentration and added as 130-μL aliquots to each well of immobilized AtAPY1-GFP on the GFP-multiTrap plate. The reaction was incubated under shaking (500 rpm) at 30°C for 1 h. The released phosphate was assayed by transferring 60 μL of each reaction mixture to two separate wells on a new transparent 96-well microtiter plate (Greiner Bio-One, Kremsmünster, Austria) and by adding 120 μL of freshly prepared stopping solution of 0.375 M H_2_SO_4_, 0.75% (w/v) (NH_2_)_4_MoO_4_ · 4H_2_O, 0.7% (w/v) SDS and 3% (w/v) FeSO_4_ · 7H_2_O to each well. After a 10-min incubation at RT, the absorbance of the samples was read at 740 nm. To determine the background from phosphate contaminations and unspecific phosphatase activities, the reactions were run in parallel with WT protein extracts. The background absorbance readings were subtracted from the readings assayed with AtAPY1-GFP.

### Solubilization of microsomal membrane proteins

Seedlings from 2 mg of *35S::AtAPY1*-GFP transgenic seeds were grown in 60 mL of liquid medium for two weeks and then ground in liquid nitrogen. The plant powder was suspended in 3 mL ice-cold protein extraction buffer (50 mM HEPES KOH pH 6.5, 5 mM EDTA, 0.4 M sucrose, 1 mM AEBSF (4-(2-aminoethyl)-benzensulfonyl fluoride hydrochloride) (Sigma), complete EDTA-free protease inhibitor cocktail (Roche)). AEBSF and the inhibitor cocktail were added right before use. The protein suspension was filtered through a single layer of miracloth and centrifuged at 14,000 *g* for 10 min at 4°C. The supernatant was ultracentrifuged at 100,000 *g* for 1 h at 4°C to pellet microsomal membranes. Equal amounts of the membranes were either treated with 2 M NaCl, 0.2 M Na_2_CO_3_, 0.2% (w/v) SDS, 4 M urea or protein extraction buffer alone for 30 min on ice. Then, the samples were centrifuged at 100,000 *g* for 1 h at 4°C producing a supernatant with solubilized proteins (S100) and the microsomal membrane fraction (P100). The supernatants were centrifuged in Vivaspin 2 concentrators (polyethersulfone membrane, 10-kDa cut off; Sartorius) for circa 20 min at 12,000 *g*. The protein concentrations of the S100 and P100 fractions were determined with the BCA protein assay kit (Thermo Scientific). Equal amounts (approximately 40 μg) of the membrane fraction and of the solubilized proteins were loaded on a SDS gel and immunoblotted.

### SDS-PAGE and Western blot analysis

The SDS-PAGE and semidry immunoblotting procedures were performed according to standard protocols. The nitrocellulose membrane (Schleicher & Schüll) was blocked in 1% (w/v) skim milk for 1 h. Primary and secondary antibodies were diluted with 1% (w/v) skim milk in TBS. After incubation of the membrane with the antibodies for 1 h each, it was washed three times for 10 min each with 0.1% (v/v) Tween-20 in TBS. AtAPY1-GFP was detected with monoclonal mouse α-GFP (1:1000; Roche), actin with monoclonal mouse α-plant actin 8 (clone 10-B3 MAbGPa; 1:1000; Sigma), cFBPase with polyclonal rabbit α-*A. thaliana* cFBPase (1:1000; Agrisera). The secondary monoclonal goat antibodies α-mouse IgG and α-rabbit IgG, both conjugated with HRP (GE Healthcare), were diluted 1:5000. The ECL Western blotting reagents (GE Healthcare) were used for the chemiluminescent signal detection.

### Accession numbers

*AtAPY1* [TAIR:At3g04080]*, AtAPY2* [TAIR:At5g18280], *Got1p homolog* [TAIR:At3g03180], *MEMB12* [TAIR:At5g50440], *Rab E1d* [TAIR:At5g03520], *SPIK* [TAIR:At2g25600], *SYP32* [TAIR:At3g24350].

## Results

### Rescue of the seedling-lethal apyrase double knockout phenotype with tagged AtAPY1

One objective was to localize AtAPY1 at the subcellular level to learn how the protein exerts its function in plant growth. Tagging AtAPY1 was chosen over raising antibodies against it because AtAPY1 and AtAPY2 are so identical in their amino acid (aa) sequence: There is only one six-aa-stretch in AtAPY1 (aa 44–49) that has four different and two similar aa to the corresponding sequence in AtAPY2 [43]. All other stretches of differences between the two sequences comprise only one or two aa.

Among the tags available, the SNAP-tag [44,45] seemed the most suitable. As an O^6^-alkylguanine-DNA alkyltransferase, SNAP binds covalently to benzylguanine-based substrates. There are a large number of substrates coupled to different fluorescent dyes and other labels commercially available making the SNAP-tag a versatile tool for localization studies. The expression of *AtAPY1-SNAP* was placed under the control of the native promoter region, because overexpression can lead to localization artifacts. Despite this risk, another tagged AtAPY1 version, AtAPY1-GFP, was fused to the strong cauliflower mosaic virus *35S* promoter because expression levels of NTPDases are generally low [46].

The SNAP- or GFP-tag was fused to the C-terminus of AtAPY1 to avoid losing the tag by a possible N-terminal cleavage in a subcellular targeting process. Since tags can impair protein function and lead to mislocalization [47], a complementation strategy was performed. The knockout of *AtAPY1* and *AtAPY2* (DKO) is seedling-lethal [18]. A DKO seedling should survive if it is complemented with a tagged AtAPY1 that is functional and correctly localized. However, the use of the *35S* promoter made the rescue of the DKO mutant with AtAPY1-GFP impossible as confirmed experimentally, because this promoter is turned off in pollen [48]. Without AtAPY1-GFP in the DKO pollen, no progeny will form, because the presence of either AtAPY1 or AtAPY2 is prerequisite for pollen to germinate [17]. In order to overcome this hurdle, partially complemented apyrase *apy2* SKO plants (= *+/apy1; apy2/apy2*; *SPIK::AtAPY2*) were used as the genetic background for transformation with each tagged *AtAPY1* construct. These plants carried *AtAPY2* under the control of the pollen-specific promoter *SPIK* which ensured the survival of the DKO pollen.

**Figure 1:**
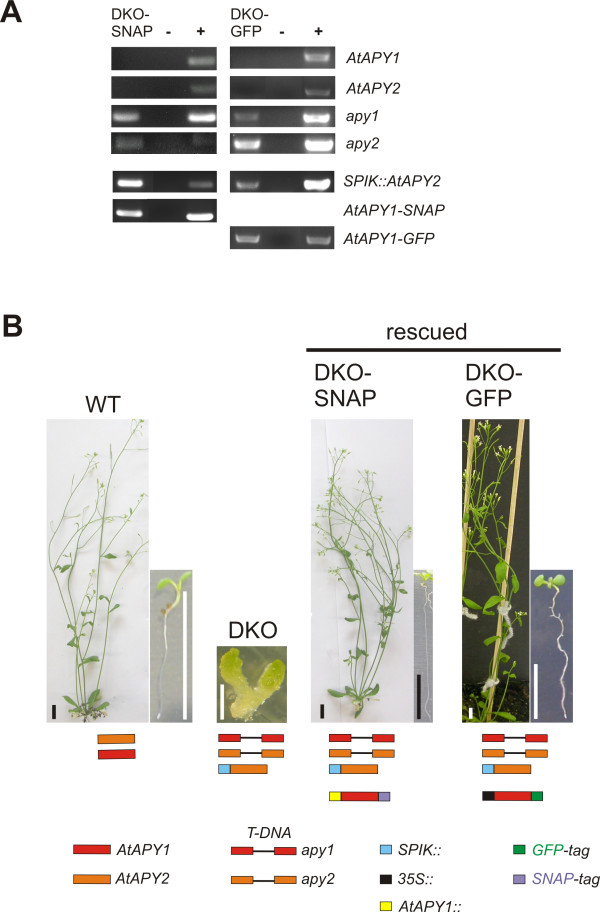
**Identification of apyrase double knockouts complemented with *AtAPY1::AtAPY1-SNAP *or *35S::AtAPY1-GFP. *(A)** Genomic DNA was isolated from DKO plants complemented with either *AtAPY1::AtAPY1-SNAP* (DKO-SNAP) or *35S::AtAPY1-GFP* (DKO-GFP) and subjected to PCR analysis. The PCR products for *AtAPY1* (1.0 kb), *AtAPY2* (0.9 kb), the T-DNA null mutations *apy1* (0.7 kb) and *apy2* (0.6 kb), *AtAPY1-SNAP* (0.6 kb), *AtAPY1-GFP* (0.7 kb) and *SPIK::AtAPY2* (0.4 kb) were analyzed by agarose gel electrophoresis. Genomic DNA from plants which had been tested positive for the respective amplification products before served as the positive controls (+). The negative PCR controls (−) were run without addition of any DNA template. **(B)** The phenotype of the WT, the DKO and the DKO rescued with either the SNAP- or GFP-tagged AtAPY1 are shown. The genetic backgrounds are represented by colored symbols. Images of 8-d- (WT), 14-d- (DKO), 7-d- (DKO-SNAP), 7-d-old (DKO-GFP) seedlings and of 30-d-old adult plants were taken. Scale bars equal 1 cm.

DNA was isolated from progeny of the partially complemented SKO plants containing either *AtAPY1::AtAPY1-SNAP* or *35S::AtAPY1-GFP* and used for genotyping by PCR. Several DKO plants without a WT *AtAPY1* and *AtAPY2* gene, but with a tagged apyrase construct were identified hereafter called DKO-SNAP and DKO-GFP, respectively. The PCR analysis of two such mutants is shown in Figure 1A. The *SPIK::AtAPY2* construct was always present in the DKO-GFP mutants as expected, but interestingly also in the DKO-SNAP mutants. One possible explanation is that some regulatory elements necessary for optimal expression in pollen were missing in the chosen promoter region. The promoter region used previously for *AtAPY1::GUS* analyses [12,17,18] was 1 kb longer at the 3’end including almost the entire gene (At3g04090) upstream of *AtAPY1*. Since the gene At3g04090 was deemed unnecessary for successful complementation, it was mostly excluded in the *AtAPY1::AtAPY1-SNAP* construct.

To confirm that the SNAP- and GFP-tagged AtAPY1 could rescue the lethal DKO seedling phenotype, the seedling phenotype of complemented SKO and DKO plants in comparison with the WT and DKO seedlings was analyzed (Figure 1B). SKO-SNAP (*+/+; apy2/apy2*; *SPIK::AtAPY2; AtAPY1::AtAPY1-SNAP*) and SKO-GFP (*apy1/apy1; +/+*; *SPIK::AtAPY2; 35S::AtAPY1-GFP*) plants were included in the study to check for possible dominant negative effects of the tagged apyrase on the WT phenotype. DKO seedlings without a construct coding for a tagged AtAPY1 had an abnormal phenotype with fleshy cotyledons and no root (Figure 1B; [18]). These seedlings did not develop beyond this stage. DKO plants expressing *AtAPY1-SNAP* or *AtAPY1-GFP*, on the other hand, showed no phenotypical differences to WT plants (Figure 1B) and SKO mutants (data not shown).

The lethal DKO (*apy1/apy1; apy2/apy2; SPIK::AtAPY2)* could be rescued by transformation with *AtAPY1*-*SNAP* or *AtAPY1-GFP* making the DKO-SNAP and DKO-GFP plants suitable tools for localization studies.

### AtAPY1 is present in punctate structures, but not at the plasma membrane or extracellular space

For localization of AtAPY1 at the subcellular level by confocal microscopy, living DKO-SNAP seedlings were incubated with SNAP-compatible fluorescent substrates to label the AtAPY1-SNAP fusion protein. Two cell-permeable, fluorescent substrates were used: red fluorescent tetramethylrhodamine-Star and the green fluorescent BG-505 (both kindly provided by Andreas Brecht, formerly Covalys Biosciences, Basel, Switzerland). Although specific labeling of fusion proteins *in vivo* was successful in yeast [49] and animal as well as human cell cultures [50-52], a high background made the detection of AtAPY1-SNAP-specific signals in Arabidopsis seedlings impossible. The tested dyes passed the cell wall and entered the cell, but even 14-h washing steps could not remove the excess fluorescent substrate (data not shown).

**Figure 2:**
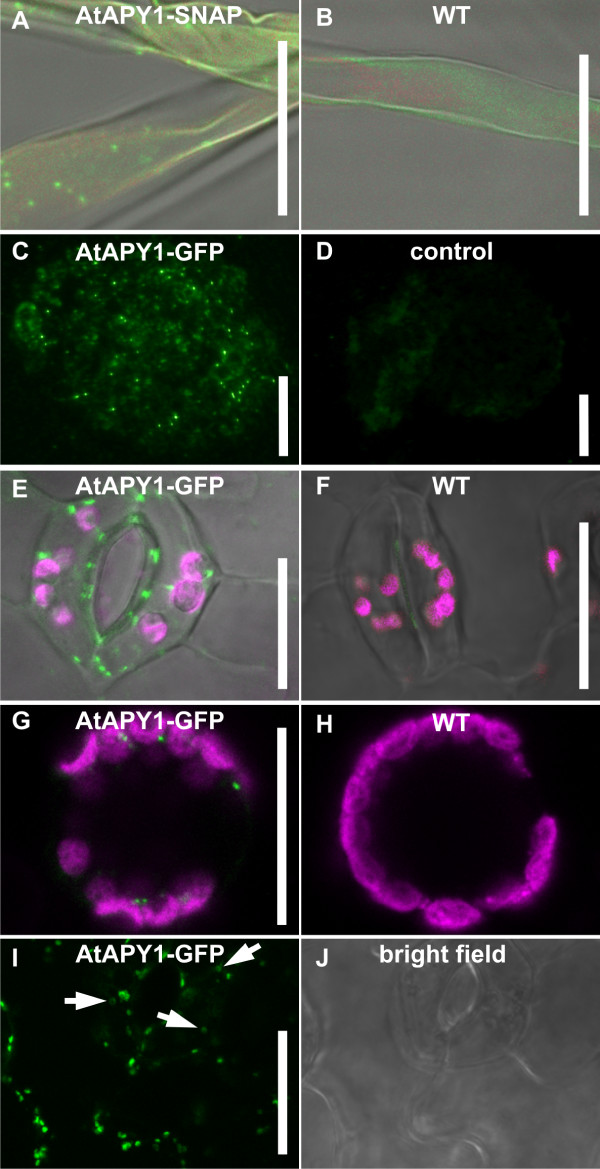
**AtAPY1 is present in intracellular dot-like structures, but absent from the extracellular space.** CLSM (**A**, **B**, **E-J**) and fluorescence microscopy (**C**, **D**) images of various cell types are depicted. All WT control images were captured using the identical CLSM settings as for the corresponding images of the transgenic plants. The FITC, Alexa Fluor 488 and GFP fluorescence is shown in green, the chlorophyll autofluorescence in magenta. The fluorescence signals are overlaid with a bright field image in **A, B**, **E** and **F**. Scale bars = 20 μm. **(A, B)** Root hairs of DKO-SNAP and WT seedlings were fixed and successively incubated with α-SNAP antibodies and secondary α-rabbit IgG coupled with horseradish peroxidase. FITC tyramides were added to amplify the fluorescence signal. The parameters of the indirect immunofluorescence detection were modified in multiple independent experiments until FITC-specific fluorescence signals as shown here were obtained. **(C, D)**Two hundred-nm Tokuyasu cryo-sections through root tips of *AtAPY1-GFP* expressing seedlings were fixed and incubated with **(C)** or without **(D)** α-GFP antibodies. All samples were incubated with secondary α-rabbit Fab fragments coupled with Alexa Fluor 488. The pictures **(C)** and **(D)** were taken with the same exposure times. **(E, F)** Two guard cells of a *35S::AtAPY1-GFP* transgenic and WT seedling are depicted. The dot-like green fluorescent signals were obtained in at least 20 independent live imagings. **(G)** A protoplast expressing *AtAPY1-GFP* and a WT protoplast **(H)**, both prepared from cotyledons, were imaged. Overlays of the green and magenta fluorescent signals are shown. These images represent the results from three independent protoplastations. **(I, J)** The GFP fluorescence and the bright field image of the same epidermal section of a cotyledon from an *AtAPY1-GFP* overexpressing seedling grown under alkaline conditions are shown representative of the imaging results from three seedlings. Doughnut- or horseshoe-shaped fluorescent structures typical of Golgi stacks imaged from the top are indicated by white arrows.

Therefore, indirect immunofluorescence was chosen as a different approach. DKO-SNAP seedlings were fixed. After cell wall digestion and plasma membrane permeabilization, they were incubated with primary antibodies against the SNAP-tag. Following several washing steps, FITC-labeled secondary antibodies were added to visualize AtAPY1-SNAP for the CLSM. The background was low, but no specific signals could be detected (data not shown). To increase the fluorescent signal, the tyramide signal amplification (TSA) technique was applied [53]. This technique employs peroxidase activity to covalently couple a large number of labeled substrates in the immediate vicinity. Therefore, instead of FITC-labeled secondary antibodies, HRP-labeled antibodies were added in combination with FITC-coupled tyramides. TSA improved the signal-to-noise ratio and intracellular dot-like structures became visible in DKO-SNAP seedlings (Figure 2A) which were not found in the WT (Figure 2B). Root hairs were selected as suitable cell types for localization, because promoter-glucuronidase analyses suggested that *AtAPY1* is expressed strongly in root hairs as well as in guard cells among other cell types [12,18].

To overcome the weak expression levels of *AtAPY1*, the indirect immunofluorescence approach was repeated with transgenic plants expressing *AtAPY1*-*GFP* under the control of the strong 35S promoter. We used primary antibodies against GFP and secondary Alexa Fluor 488-coupled antibodies in two different approaches: 1. Post-embedding labeling of 200-nm-thin Tokuyasu cryo-sections (Figure 2C, D), and 2. Pre-embedding labeling followed by embedding in Technovit 7100 resin and sectioning (Additional file 3). In both experiments, the intracellular punctate signals could be confirmed in the root (Figure 2C) and in root hairs (Additional file 3). No signals were detected in the cell wall and at the plasma membrane and in the control without primary antibody (Figure 2D).

To verify the imaging data obtained by immunofluorescence, a second detection method was used. The transgenic plants expressing *AtAPY1GFP* were imaged *in vivo* by CLSM. Here, the same intracellular punctate pattern as found before in *AtAPY1-SNAP* and *AtAPY1-GFP* expressing plants was observed in guard cells (Figure 2E), cotyledon epidermis (Additional file 4A), hypocotyls (Additional file 4B) and roots (Additional file 4C). The WT control did not present this punctate pattern as shown exemplarily for WT guard cells (Figure 2F). Expression of the *GFP*-tag alone led to cytoplasmic staining [28].

The method of live imaging of GFP-tagged proteins is suitable to detect apyrase in the cell wall as shown for apoplastic apyrases in other plant species [16,54]. But since AtAPY1 was expected to be localized extracellularly and since GFP does not fluoresce at  pH ≤5.0 [55], a weak AtAPY1-GFP signal could be missed if the tag were exposed to the acidic environment of the cell wall. To provide an optimal pH for GFP fluorescence in the extracellular environment, WT protoplasts and those expressing *AtAPY1-GFP* were prepared from cotyledons and imaged at pH 7.6. As before, intracellular GFP signals were found (Figure 2G) which did not appear in the WT control (Figure 2H), but the plasma membrane of the transgenic protoplasts did not fluoresce (Figure 2H). This result ruled out the possibility that AtAPY1 was anchored in the plasma membrane. However, protoplastation represents severe stress for the cells which could have caused down regulation of *AtAPY1* and/or degradation or internalization of AtAPY1. In addition, AtAPY1 was a possibly soluble protein in the cell wall and in this case the digestion of the cell wall during protoplast preparation would have led to a loss of the AtAPY1-GFP signal. Therefore, cells with intact walls were imaged at a pH suitable for GFP fluorescence (Figure 2I, J). Seedlings expressing *35S::AtAPY1-GFP* were grown in liquid culture at pH of 8.1 instead of 5.7. The higher pH in the culture medium is known to recover GFP fluorescence in the apoplast [38]. However, even under these conditions, no extracellular GFP signal was detectable (Figure 2I). In addition, *35S::AtAPY1-GFP* seedlings were cultured under normal conditions to minimize any impact the alkaline culture medium may have on AtAPY1 distribution and infiltrated with buffer of pH 7.5 just for imaging. Again no extracellular signals were found in more than 30 independent experiments (data not shown).

Both detection methods, immunofluorescence and *in vivo* imaging, revealed the same punctate structures, but no signals at the plasma membrane or in the extracellular space.

### AtAPY1 is localized in the Golgi apparatus

**Figure 3:**
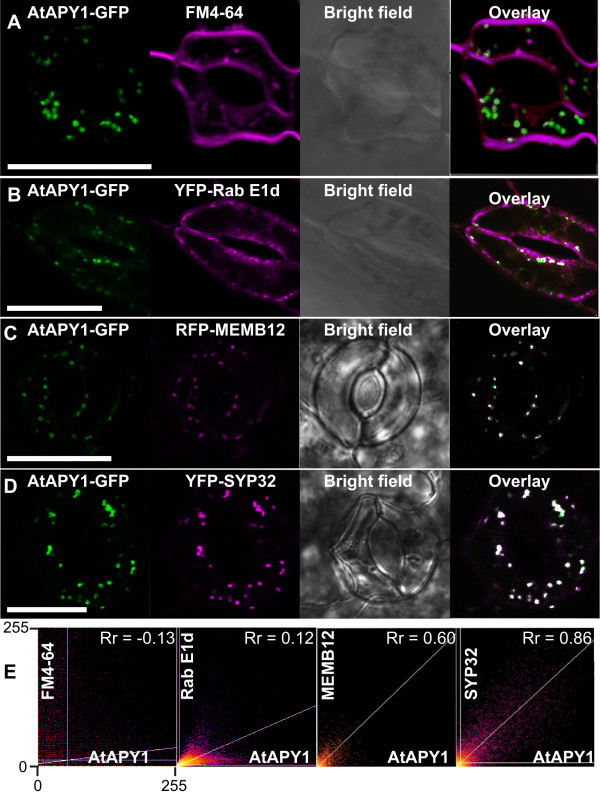
**Identification of AtAPY1-positive structures as Golgi.** CLSM images of epidermal cells from cotyledons are shown. The GFP fluorescence is shown in green, the FM4-64 **(A)**, YFP **(B, D)** and RFP **(C)** fluorescence in magenta. The images from the two fluorescence detection channels were merged with the “Co-localization Finder” plugin (= overlay) and co-localization of the green and magenta signals resulted in white spots. Scale bars = 20 μm. **(A**) Transgenic seedlings expressing *AtAPY1-GFP* were treated with 15 μM FM4-64 by vacuum-infiltration. A section of the lower epidermis is shown after 120 min of treatment. **(B)** Two guard cells of a transgenic seedling co-expressing *AtAPY1-GFP* and *YFP-Rab E1d* are shown. **(C, D)** Transgenic seedlings co-expressing *AtAPY1-GFP* and either *RFP-MEMB12* or *YFP-SYP32* were imaged. **(E)** The distribution of the green and magenta pixels in the dual-channel overlay images in **A-D** were analyzed with the ImageJ “Colocalization Threshold” and “Coloc2” tool. The x-axes represent the intensities of the green pixels from the GFP channel (AtAPY1) and the y-axes from the magenta channel (FM4-64, Rab E1d, MEMB12 or SYP32). For each scatterplot, the intensities are given as the pixel grey values ranging from 0 to 255. Co-localization clusters the pixels from both channels along a diagonal line. The maximal theoretical value for the Pearson’s correlation coefficient (R_r_) is 1.0.

At higher magnifications, some of the AtAPY1-specific punctate signals appeared as doughnut- or horseshoe-shaped structures (see Figure 2I). This morphology is typical of Golgi stacks viewed in the middle of the main cisternae from the top [56]. In addition, the observed size between 0.5 to 1 μm across matched the expected size of Golgi stacks [57]. In order to corroborate that AtAPY1 was localized in these organelles, the dye FM4-64 was applied. FM4-64 is endocytosed by the cell, sequentially staining the plasma membrane, endosomes and the *trans*-Golgi network, but not the Golgi apparatus [58]. Imaging FM4-64-infiltrated *35S::AtAPY1-GFP* seedlings did not reveal any co-localization of the fluorescent dye and the GFP signal, even 120 min after the infiltration (Figure 3A and corresponding scatterplot in Figure 3E).

As an additional negative endosomal control, co-localization with the GTPase Rab E1d was studied. There is some controversy in the literature over the designation of Rab E1d as a marker protein for the Golgi [59] or the post-Golgi/endosomal compartment [32]. However, there is consensus that Rab E1d primarily co-localizes with rat sialyltransferase [59,60], a *trans*-Golgi and/or *trans*-Golgi network marker protein [61], and that it also associates with the plasma membrane (PM) [60,62]. Therefore, Rab E1d is believed to play a role in the trafficking of secretory vesicles from the Golgi to the PM [59,60].

In transgenic plants co-expressing *YFP-Rab E1d* and *AtAPY1-GFP*, no overlap of the YFP and GFP fluorescence was found (Figure 3B and corresponding scatterplot in Figure 3E). The lack of overlap not only suggests the absence of AtAPY1-GFP in endosomes, but also ruled out crosstalk between the GFP and YFP detection channels. In order to confirm that the chosen GFP and YFP settings were specific for the detection of the GFP and YFP fluorescence, respectively, transgenic plants expressing only one of the two fluorophores were imaged sequentially with the GFP and YFP settings. When imaging epidermal cells from the *AtAPY1-GFP* expressing plant with the GFP settings, the familiar dot-like structures appeared in the GFP detection channel (Additional file 5A). Taking an image of the identical epidermal section with the YFP settings, however, did not deliver this punctate pattern (Additional file 5A). The same imaging experiment with a plant expressing *YFP-SYP32* only, showed negligible bleed-through of the YFP fluorescence into the GFP detection channel (Additional file 5B). These control experiments demonstrated the specificity of the GFP and YFP detection settings.

In a direct localization approach, the occurrence of AtAPY1-GFP in the Golgi apparatus was investigated by co-localization with three known Arabidopsis Golgi-resident proteins. MEMB12 (Membrin 12) and SYP32 (Syntaxin of plants 32) are SNARE proteins localized in the Golgi apparatus [63]. Got1p (Golgi transport 1 protein) was found in the Golgi membranes of *Saccharomyces cerevisiae*[64] and its homolog in Arabidopsis was also localized in the Golgi [32]. Transgenic plants co-expressing *AtAPY1-GFP* and either the Golgi marker *RFP-MEMB12* (Figure 3C), the *YFP-SYP32* (Figure 3D) or *YFP-Got1p homolog* (Additional file 6) were analyzed by confocal microscopy. The fluorescence of all three Golgi marker proteins overlapped with the AtAPY1 fluorescence, localizing AtAPY1-GFP to the Golgi apparatus.

To exclude or confirm a co-localization not only by eye, the ImageJ software was applied. The overlays of the images from the two detection channels were used to generate scatterplots and to calculate the Pearson’s correlation coefficient (R_r_) of the two fluorescent signals (Figure 3E and Additional file 6). R_r_ values > 0.5 indicate co-localization [65], verifying co-localization of all three marker proteins RFP-MEMB12, YFP-SYP32 and YFP-Got1p homolog with AtAPY1-GFP.

**Figure 4:**
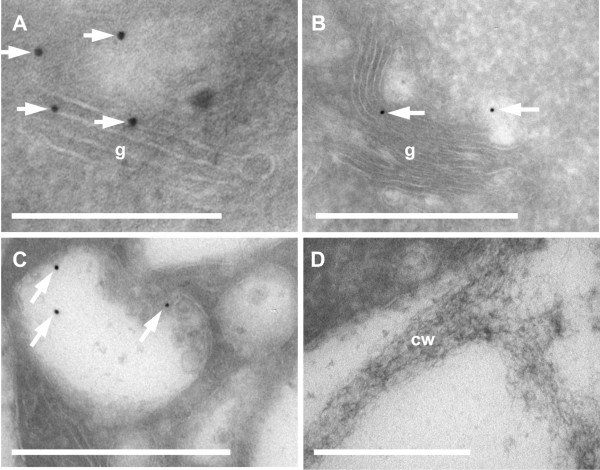
**Confirmation of the Golgi localization of AtAPY1 by immunogold labeling.** (**A**-**D**) Transverse Tokuyasu-cryo-sections through root tips were imaged by TEM after imunogold labeling of AtAPY1-GFP using α-GFP antibodies (Torrey Pines) and Protein A 10-nm gold. Arrows mark gold particles. Independent experiments with Protein A 6-nm gold gave the same localization results presented here. Both approaches were repeated three times. Abbreviations: cw, cell wall, g, golgi stack, mvb, multivesicular body. Scale bars equal 200 nm in **A** and 500 nm in **B-D**.

In order to confirm the co-localization results, AtAPY1-GFP was labeled with gold particles using α-GFP primary antibodies and secondary gold-coupled secondary antibodies. Electron microscopy of Tokuyasu cryo-sections through roots revealed weak, but specific staining of Golgi stacks (Figure 4A,B). In sections through 56 Golgi compartments (48 labeled, 8 unlabeled) equaling an area of 11.8 μm^2^, 8.3 gold particles per μm^2^ were counted. Considering only labeled Golgi compartments increased the value to 9.8 particles per μm^2^. By comparison, only 0.37 and 0.07 gold particles per μm^2^ were found in sections through 88 mitochondria (= 14 μm^2^) and 12 nuclei (= 27 μm^2^), respectively. Multivesicular bodies (MVB) were also positively immunolabeled by gold particles (Figure 4C) which most likely reflects the transport of some AtAPY1-GFP to the vacuole as seen in other *GFP*-overexpressing plants [59] rather than a functional role of AtAPY1 in this prevacuolar compartment. No immunolabeling of any other cellular compartment including the cell wall was found (Figure 4D). In summary, the immunogold labeling studies of AtAPY1-GFP confirmed its localization in the Golgi and gave no indication of an extracellular occurrence.

### AtAPY1 has the substrate specificity typical of an endo-, not an ecto-apyrase

**Figure 5:**
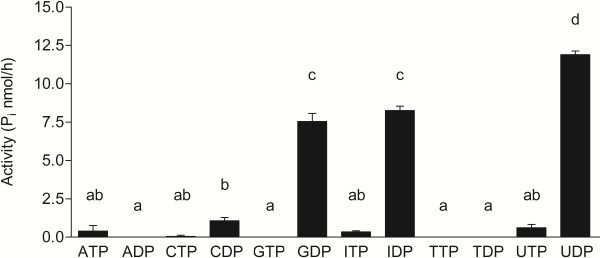
**Substrate specificity of AtAPY1-GFP. **The activity of AtAPY1-GFP in the presence of various substrates at pH 6.5 was measured. Different letters above the columns indicate mean values that are significantly different from one other (p<0.05, Tukey test). Error bars represent standard deviations of duplicate measurements from one reaction (see Methods). The data are representative of five activity assays with independent protein extracts. Abbreviation: P_i_, inorganic phosphate.

If the Golgi localization of AtAPY1 were correct, AtAPY1 should exhibit the substrate specificity typical of Golgi apyrases. Therefore, the activity of AtAPY1-GFP was tested in the presence of known apyrase substrates at pH 6.5 which equals the pH found in the Golgi [55]. AMP is not a substrate for apyrases and for this reason served as a negative control. AtAPY1-GFP did not hydrolyze ATP and ADP, the typical substrates of ecto-apyrases [46], but the nucleotides uridine diphosphate (UDP), guanosine diphosphate (GDP) and inosine diphosphate (IDP) (Figure 5). No activity was detectable in the presence of all other NTPs or NDPs (Figure 5) and AMP (data not shown). Hydrolysis of these three NDPs matches the substrate specificity of other plant Golgi apyrases, e. g. from rice (*Oryza sativa*) [66] and sycamore (*Acer pseudoplatanus*) [67].

In order to investigate the possibility that the substrate specificity was pH-dependent and that it would change in favor of ATP and ADP once AtAPY1 reached the apoplast, the activity assay of AtAPY1-GFP was repeated at pH 5.5, the pH typically found in the cell wall [68]. However, the three diphosphates UDP, GDP and IDP remained the only substrates (Additional file 7). Therefore, the determined substrate specificity substantiated the results that AtAPY1 was not observed in the cell wall, but in the Golgi.

### AtAPY1 is an integral membrane protein

**Figure 6:**
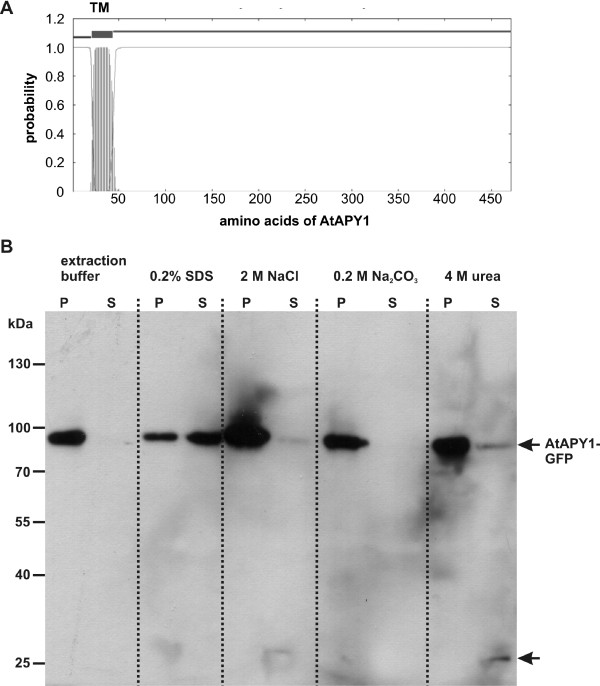
**Analysis of the solubility of AtAPY1-GFP.(A)** The output of the TMHMM prediction program [69] is shown. The AtAPY1 aa sequence is represented along the x-axis with the N-terminal aa being the first residue. The probability for each aa to appear within a transmembrane domain (TM) is given. One TM is predicted which comprises the aa 21–43. **(B**) Microsomal membranes were prepared from transgenic plants containing *35S::AtAPY1-GFP*. Equal amounts of membrane fractions were treated either with extraction buffer, 0.2% SDS, 2 M NaCl, 0.2 M Na_2_CO_3_ or 4 M urea. After a 30-min treatment, the proteins were subjected to centrifugation at 100,000 *g*. The pellet fractions (P) and the supernatants (S) were separated in a 8% SDS polyacrylamide gel, blotted on a nitrocellulose membrane and incubated with α-GFP antibodies. The molecular weights of the protein standard are given in kDa. The AtAPY1-GFP protein (80 kDa) and a second band at 27 kDa (arrow) were detected with α-mouse IgG coupled with horseradish peroxidase. Similar results were obtained in three experiments with independent protein extracts.

One objective was to determine if AtAPY1 was a soluble protein in the lumen of the Golgi or, as implied by the TMHMM prediction program [69], a Golgi membrane protein with an uncleaved signal sequence at the N-terminus serving as a transmembrane anchor (Figure 6A). This prediction was supported by the immunogold labeling results which suggested a membrane association of the protein (Figure 4A-C).

Microsomal membranes were isolated from transgenic *AtAPY1-GFP* seedlings and their purity was verified with antibodies against marker proteins for cytosolic and insoluble proteins (Additional file 8). The microsomal membranes were treated with various solubilizing agents and then analyzed for any solubilized proteins. Using α-GFP antibodies, a major signal was observed at the expected molecular mass of AtAPY1-GFP (80 kDa) and a minor signal at 27 kDa (Figure 6B). The smaller protein most likely represents a degradation product of AtAPY1-GFP resulting from the protein extraction procedure. If the microsomal membranes were left untreated, AtAPY1-GFP was detected in the membrane fraction (Figure 6B), suggesting that the protein was membrane-bound. In support of this finding, the detergent SDS released the majority of the AtAPY1-GFP protein from the membranes (Figure 6B). In order to differentiate between AtAPY1 being a peripheral or an integral membrane protein, the microsomal membranes were subjected to high salt (2 M NaCl), alkaline (0.2 M Na_2_CO_3_) and denaturing (4 M urea) conditions. Peripheral proteins are removed from membranes by urea which disturbs protein-protein interactions or by high salt and alkaline treatments which disrupt electrostatic and hydrophobic interactions, respectively. Except for trace amounts, the salt and Na_2_CO_3_ treatment did shift AtAPY1 from the pellet fraction to the supernatant (Figure 6B) as expected for an integral membrane protein. AtAPY1 is also not a Golgi soluble protein, because AtAPY1 remained in the pellet fraction after Na_2_CO_3_ treatment which is known to leach the soluble proteins from the microsomal lumen into the supernatant fraction as shown in [70]. Urea released some AtAPY1-GFP protein into the supernatant, but most of the protein remained in the membrane fraction (Figure 6B). Although transmembrane proteins are generally not extracted by urea at all, type II integral proteins seem to be less tightly associated with the membrane than proteins with multiple transmembrane domains [71].

In summary, AtAPY1 showed the characteristics of a single-pass type II membrane protein.

## Discussion

### Previous evidence for extracellular localization of AtAPY1

Using programs to predict the subcellular localization of AtAPY1 was inconclusive: The program SubLoc version (v) 1.0 [72] suggested the cytoplasm, Target P v. 1.1 [73] mitochondria and WoLF PSORT v. 2.0 [74] the chloroplast. Predotar v. 1.3 [75] could not define a specific localization and PSORT v. 6.4 [76] predicted an extracellular localization. Therefore, experimental data became invaluable. Experiments with *in vitro* germinated pollen detected apyrase activity in the liquid germination medium [12]. This extracellular activity was inhibited by the addition of polyclonal antibodies raised against the N-terminally truncated (aa 36–471), denatured and recombinant AtAPY1 protein [12]. This result indicated that AtAPY1 was an extracellular protein. However, these antibodies might have targeted apyrases other than AtAPY1 in the cell wall. The apyrase conserved region (ACR) 1 of AtAPY1, for example, is 100% identical to the ACR1 of AtAPY2. Therefore, it is not surprising that the α-AtAPY1 antibodies also recognize AtAPY2 [12]. The similarity of the AtAPY1 ACRs with the other five Arabidopsisapyrases is not as high, but these conserved regions could still be binding targets for the α-AtAPY1 antibodies. The possibility of cross-reactivity is supported by experiments using the same α-AtAPY1 antiserum to successfully inhibit extracellular apyrase activity in a different plant species, namely cotton [14].

The experiment with the *in vitro* germinated pollen also showed that the protein exhibiting apyrase activity in the pollen germination medium was soluble because it was measureable in siphoned off aliquots [12]. Since AtAPY1 showed the solubilization characteristics of an integral membrane protein, it is unlikely to be identical to the previously detected extracellular apyrase activity.

### Arabidopsis apyrases as regulators of eATP signals

AtAPY1 and AtAPY2 have been implicated to be the regulators of eATP signals in Arabidopsis [12,14,15,77-79] and were therefore expected to function in the cell wall. However, the substrate specificity of AtAPY1 does not fit this model. AtAPY1-GFP did not hydrolyze ATP and ADP, the typical substrates of ecto-apyrases, contradicting a role of AtAPY1 in regulating eATP signals.

Even though AtAPY1 did not agree with the profile of an ecto-apyrase, AtAPY2 remains a candidate. However, Dunkley et al*. *[80] noted a Golgi localization of AtAPY2. They prepared membrane fractions from Arabidopsis callus cultures by density centrifugation and identified AtAPY2 in the Golgi protein pool by mass spectrometry. Recently, the Golgi localization of AtAPY2 was confirmed in another proteome analysis of enriched Golgi membranes [81]. Nevertheless, the Arabidopsis Information Resource database holds entries for two different splice variants of *AtAPY2* that could result in different localizations of the corresponding proteins. The fact that transgenic Arabidopsis plants overexpressing *AtAPY2* show less eATP-mediated superoxide production than WT [79] points to an extracellular localization of (a variant of) AtAPY2.

Since AtAPY1 was not detected in the cell wall by three subcellular detection methods and did not exhibit the substrate specificity of an ecto-apyrase, we hypothesize that some apyrase other than AtAPY1 is the regulatory enzyme observed in eATP signaling of Arabidopsis.

### Possible AtAPY1 function(s) in the Golgi apparatus

A diphosphatase activity was described in the Golgi of *Pisum sativum*[82]. UDP was rapidly degraded to uridine monophosphate and P_i_ by an integral membrane protein facing the Golgi lumen with its active site. It was also shown in *P. sativum* that UDP inhibits glycosyltransferases [83], which function in the assembly of primary plant cell wall components [84] in a feedback mechanism. So it was proposed that a diphosphohydrolase such as apyrase prevents inhibition of polysaccharide synthesis in the Golgi by constantly removing the by-product UDP from the glycosyl transfer reaction [85]. A role of the Golgi apyrase in polysaccharide biosynthesis was supported by their finding that the enzyme was only found in the elongation zone of the pea seedling stem. This zone constantly needs new cell wall material. The apyrase AtAPY1 could assume the same role in Arabidopsis. The developmental and growth phenotypes of the apyrase DKO mutants such as lack of pollen germination [17], no root and shoot growth, and distorted cell shapes [18] could be explained by defects in the biosynthesis of cell wall material. Such a role is supported by the substrate specificity of AtAPY1 because UDP and GDP are produced by glycosyltransferases in the Golgi.

xMore evidence for a function in glycosylation comes from a complementation experiment with the yeast *Saccharomyces cerevisiae* mutant *Δgda1*. This yeast mutant lacks the Golgi apyrase guanosine diphosphatase 1 (GDA1) leading to a glycosylation defect [86] which is abolished by complementation with *AtAPY1*[81].

It is also believed that Golgi apyrases provide the nucleoside monophosphates which serve as the substrates in exchange for nucleotide sugars from the cytosol in an antiport mechanism [85]. Therefore, a shortage of nucleoside monophosphates would prevent or reduce the transport of nucleotide sugars into the Golgi [87] and increase the nucleotide sugar concentration in the cytosol. As a “release valve”, the chloroplasts would import the sugar and convert it into starch. In agreement with this hypothesis, a pronounced increase in transitory starch was observed in the apyrase DKO mutants compared with the WT [18]. An accumulation of starch in the chloroplasts was also seen when the Golgi function was generally disrupted and explained by an indirect effect due to elevated cytosolic sugar concentrations [88].

## Conclusions

The use of two different transgenic lines expressing either *AtAPY1-SNAP* or *-GFP* allowed specific labeling of AtAPY1 at the subcellular level. The cell organelles exhibiting the AtAPY1-specific fluorescent signals were identified as the Golgi apparatus by the following criteria: (i) morphology, (ii) size, (iii) lack of co-localization with the endocytic marker stain FM4-64 and *trans*-Golgi marker protein Rab E1d, but (iv) co-localization with the three Golgi marker proteins MEMB12, SYP32 and Got1p homolog. In addition, Golgi stacks were immunolabeled with α-GFP antibodies. While this paper was under review, the Golgi localization of AtAPY1 was independently confirmed by proteome analysis of Golgi membranes and by co-localization of AtAPY1-YFP with the CFP-labeled *cis*-Golgi marker α-mannosidase I in transiently transformed onion peels [81].

Although it can never be ruled out that the missing detection of extracellular AtAPY1 is a matter of methodological sensitivity, our results show that AtAPY1 is primarily present in the Golgi. Therefore, AtAPY1 is highly unlikely to represent the regulatory enzyme of eATP levels [12,14,15,77,78] and the true identity of the Arabidopsis ecto-apyrase(s) is yet to be found. Furthermore, the growth defects caused by the absence [18] and by the reduced amounts of AtAPY1 and AtAPY2 [12] are probably not directly linked to eATP signaling. Instead, the localization of AtAPY1 in the Golgi needs to be the basis for future investigations to understand how AtAPY1 in particular and plant Golgi apyrases in general affect plant growth and development.

## Abbreviations

aa: Amino acid(s); ACR: Apyrase conserved region; AEBSF: 4-(2-aminoethyl)-benzensulfonyl fluoride hydrochloride; apyrase: Adenosine pyrophosphatase; CLSM: Confocal laser scanning microscopy; DKO: Double knockout; eATP: Extracellular ATP; ECM: Extracellular matrix; FITC: Fluorescein isothiocyanate; FM4-64: N-(3-triethylammoniumpropyl)-4-(p-diethylaminophenyl-hexatrienyl)-pyridinium dibromide; GDP: Guanosine diphosphate; GFP: Green fluorescennt protein; Got1p: Golgi transport 1 protein; HeNe: Helium-neon; HRP: Horseradish peroxidase; IDP: Inosine diphosphate; MC: Methyl cellulose; MEMB12: Membrin 12; MS: Murashige and Skoog; MVB: Multivesicular body; NTPDase: Nucleoside triphosphate diphosphohydrolase; ORF: Open reading frame; P: Pellet; PB: Phosphate buffer; PBS: Phosphate buffered saline; PFA: Paraformaldehyde; P_i_: Inorganic phosphate; PM: Plasma membrane; PPT: Phosphinothricin; RFP: Red fluorescent protein; RT: Room temperature; S: Supernatant; SNAP: O^6^-alkylguanine-DNA alkyltransferase; SPIK: Shaker pollen inward K^+^ channel; SYP32: Syntaxin of plants 32; SKO: Single knockout; T-DNA: Transfer DNA; TBS: Tris buffered saline; TEM: Transmission electron microscopy; TM: Transmembrane domain; TSA: Tyramide signal amplification; UA: Uranyl acetate; UDP: Uridine diphosphate; v: Version; WT: Wild type or wild-type; YFP: Yellow fluorescent protein.

## Authors’ contributions

MS carried out the molecular genetic studies of the DKO-SNAP mutants, performed the localization studies including confocal laser scanning microscopy and image analysis, sample preparations and treatments, analyzed the solubility of the AtAPY1-GFP and participated in the drafting of the manuscript. CM performed the substrate specificity analyses of AtAPY1-GFP. TK conducted the localization studies by immunogold labeling and by indirect immunofluorescence with *AtAPY1-GFP* expressing seedlings. IS conceived of the study, generated the *AtAPY1-SNAP* transgenic lines and the lines co-expressing *AtAPY1-GFP* and a marker gene, performed the genetic complementation of the DKO-GFP mutants and drafted the manuscript. All authors read and approved the final manuscript.

## Supplementary Material

Additional file 1 ***AtAPY1-SNAP DNA sequence.*** The *AtAPY1-SNAP* DNA sequence present in the *AtAPY1::AtAPY1-SNAP* transgenic lines is shown. Click here for file

Additional file 2 ***AtAPY1-GFP DNA sequence.*** The *AtAPY1-GFP* DNA sequence present in the *35S::AtAPY1-GFP* transgenic lines is shown. Click here for file

Additional file 3 **Immunofluorescence of pre-imbedding labeled AtAPY1-GFP in root hair.** Roots from *AtAPY1-GFP* expressing seedlings were immunostained whole mount, embedded in resin and sectioned. The sections were successively incubated with α-GFP and secondary α-rabbit Fab fragments coupled with Alexa Fluor 488. A 3-μm cross-section of a root hair is shown. The Alexa Fluor 488 fluorescence is shown in green. Scale bar equals 20 μm. Click here for file

Additional file 4 **Live imaging of AtAPY1-GFP in various cell types.** CLSM images of various tissues in *AtAPY1-GFP* expressing seedlings show the GFP signals in green overlaid with bright field view. **(A)** Cotyledon epidermis, **(B)** hypocotyl and **(C)** root tip. Scale bars = 20 μm. Click here for file

Additional file 5 **Specificity of the imaging settings for the detection of GFP and YFP fluorescence.** The epidermis of cotyledons from transgenic plant lines was imaged with the GFP and YFP settings outlined under Methods for the “CLSM”. The GFP and YFP fluorescence is shown in green and magenta, respectively. Scale bars = 20 μm. **(A)** The identical epidermal section with two guard cells from plants expressing *AtAPY1-GFP* only was imaged sequentially with the GFP and YFP settings. Dot-like signals appeared in the GFP detection channel only. Only weak autofluorescence of the thickened cell wall around the stomate was visible in the YFP detection channel. **(B)** The identical epidermal section with two guard cells from plants expressing *YFP-SYP32* only was imaged sequentially with the GFP and YFP settings. Here, only very weak signals were detectable in the GFP detection channel, but strong YFP fluorescence appeared with the YFP-specific excitation and detection. Click here for file

Additional file 6 **Co-localization analysis of AtAPY1-GFP and YFP-Got1p homolog.** CLSM images of epidermal cells of cotyledons from transgenic lines co-expressing *AtAPY1-GFP* and *YFP-Got1 phomolog* were taken. The GFP fluorescence is shown in green and the YFP fluorescence in magenta. The bright field-type image was acquired with the transmitted light detector. The fluorescence signals for AtAPY1-GFP and YFP-Got1p homolog were detected separately and merged for co-localization with the “Co-localization Finder” plugin of ImageJ. Co-localization of the two proteins is depicted as white signals. The corresponding scatterplot was analyzed with the ImageJ “Colocalization Threshold” and “Coloc2” tool from ImageJ. The x-axis represents the pixel intensities from the GFP channel and the y-axis from the YFP channel. *R*_r_ *=* Pearson’s correlation coefficient*.* Scale bar = 20 μm. Click here for file

Additional file 7 **Substrate specificity of AtAPY1-GFP at pH 5.5.** The activity of AtAPY1-GFP at pH 5.5 in the presence of various substrates was measured. No activity was detectable with AMP as substrate (data not shown). Different letters above the columns indicate mean values that are significantly different from one other (p<0.05, Tukey test). Error bars represent standard deviations of two phosphate measurements from one reaction (see Methods). Abbreviation: P_i_, inorganic phosphate. Click here for file

Additional file 8 **Analysis of the purity of microsomal membrane and soluble protein fractions.** Protein extracts from transgenic plants expressing *35S::AtAPY1-GFP* were treated with either extraction buffer, 0.2% SDS, 2 M NaCl, 0.2 M Na_2_CO_3_ or 4 M urea and then centrifuged at 100,000 *g* to obtain microsomal membrane fractions (P100) and supernatants (S100). Proteins from each fraction (40 μg each) were subjected to Western blot analysis. The enrichment of microsomal and insoluble proteins in the P100 fractions and of soluble proteins in the S100 fractions was confirmed with antibodies against marker proteins. The 37-kDa cytosolic fructose-1,6-bisphosphatase (cFBPase) served as a marker protein for soluble proteins. Actin (45 kDa) was used as a marker protein for insoluble proteins under actin polymerizing conditions found in the extraction buffer and 2 M NaCl [89] and as a soluble marker under actin depolymerizing conditions such as 0.2 M Na_2_CO_3_, 0.2% SDS and 4 M urea [89,90]. Click here for file

## References

[B1] Meyerhof O The origin of the reaction of Harden and Young in cell-free alcoholic fermentation J Biol Chem 1945 157 105 119

[B2] Zimmermann H Beaudoin AR Bollen M Goding JW Guidotti G Kirley TL Robson SC Sano K Vanduffel L, Lemmens R Proposed nomenclature for two novel nucleotide hydrolyzing enzyme families expressed on the cell surface Ecto-ATPases and related ectonucleotidases 2000 Maastricht: Shaker Publishing BV 1 8

[B3] Komoszynski M Wojtczak A Apyrases (ATP diphosphohydrolases, EC 3.6.1.5): function and relationship to ATPases Biochim Biophys Acta 1996 1310 233 241 10.1016/0167-4889(95)00135-2 8611638

[B4] Handa M Guidotti G Purification and cloning of a soluble ATP-diphosphohydrolase (Apyrase) from potato tubers (Solanum tuberosum) Biochem Biophys Res Commun 1996 218 916 923 10.1006/bbrc.1996.0162 8579614

[B5] Etzler ME Kalsi G Ewing NN Roberts NJ Day RB Murphy JB A nod factor binding lectin with apyrase activity from legume roots Proc Natl Acad Sci USA 1999 96 5856 5861 10.1073/pnas.96.10.5856 10318974 PMC21950

[B6] Day R McAlvin C Loh J Denny R Wood T Young N Stacey G Differential expression of two soybean apyrases, one of which is an early nodulin Mol Plant-Microbe Interact 2000 13 1053 1070 10.1094/MPMI.2000.13.10.1053 11043467

[B7] Cohn J Uhm T Ramu S Nam Y-W Kim D-J Penmetsa R Wood T Denny R Young N Cook D Stacey G Differential regulation of a family of apyrase genes from Medicago truncatula Plant Physiol 2001 125 2104 2119 10.1104/pp.125.4.2104 11299390 PMC88866

[B8] McAlvin C Stacey G Transgenic expression of the soybean apyrase in Lotus japonicus enhances nodulation Plant Physiol 2005 137 1456 1462 10.1104/pp.104.055939 15793071 PMC1088334

[B9] Govindarajulu M Kim SY Libault M Berg RH Tanaka K Stacey G Taylor CG GS52 ecto-apyrase plays a critical role during soybean nodulation Plant Physiol 2009 149 994 1004 19036836 10.1104/pp.108.128728 PMC2633840

[B10] Thomas C Rajagopal A Windsor B Dudler R Lloyd A Roux S A role for ecto-phosphatase in xenobiotic resistance Plant Cell 2000 12 519 533 10760241 10.1105/tpc.12.4.519 PMC139850

[B11] Thomas C Sun Y Naus K Lloyd A Roux S Apyrase functions in plant phosphate nutrition and mobilizes phosphate from extracellular ATP Plant Physiol 1999 119 543 551 10.1104/pp.119.2.543 9952450 PMC32131

[B12] Wu J Steinebrunner I Sun Y Butterfield T Torres J Arnold D Gonzalez A Jacob F Reichler S Roux SJ Apyrases (nucleoside triphosphate-diphosphohydrolases) play a key role in growth control in Arabidopsis Plant Physiol 2007 144 961 975 10.1104/pp.107.097568 17434987 PMC1914212

[B13] Yuo T Toyota M Ichii M Taketa S Molecular cloning of a root hairless gene rth1 in rice Breed Sci 2009 59 13 20 10.1270/jsbbs.59.13

[B14] Clark G Torres J Finlayson S Guan X Handley C Lee J Kays JE Chen ZJ Roux SJ Apyrase (nucleoside triphosphate-diphosphohydrolase) and extracellular nucleotides regulate cotton fiber elongation in cultured ovules Plant Physiol 2010 152 1073 1083 10.1104/pp.109.147637 20018604 PMC2815863

[B15] Clark G Wu M Wat N Onyirimba J Pham T Herz N Ogoti J Gomez D Canales AA Aranda G Both the stimulation and inhibition of root hair growth induced by extracellular nucleotides in Arabidopsis are mediated by nitric oxide and reactive oxygen species Plant Mol Biol 2010 74 423 435 10.1007/s11103-010-9683-7 20820881

[B16] Riewe D Grosman L Fernie AR Wucke C Geigenberger P The potato-specific apyrase is apoplastically localized and has influence on gene expression, growth and development Plant Physiol 2008 147 1092 1109 10.1104/pp.108.117564 18480378 PMC2442552

[B17] Steinebrunner I Wu J Sun Y Corbett A Roux SJ Disruption of apyrases inhibits pollen germination in Arabidopsis Plant Physiol 2003 131 1638 1647 10.1104/pp.102.014308 12692323 PMC166920

[B18] Wolf C Hennig M Romanovicz D Steinebrunner I Developmental defects and seedling lethality in apyrase AtAPY1 and AtAPY2 double knockout mutants Plant Mol Biol 2007 64 657 672 10.1007/s11103-007-9184-5 17534719

[B19] Alvarado-Castillo C Lozano-Zarain P Mateo J Harden TK Boyer JL A fusion protein of the human P2Y(1) receptor and NTPDase1 exhibits functional activities of the native receptor and ectoenzyme and reduced signaling responses to endogenously released nucleotides Mol Pharmacol 2002 62 521 528 10.1124/mol.62.3.521 12181428

[B20] Burnstock G Purinergic signalling Br J Pharmacol 2006 147 Suppl 1 S172 S181 16402102 10.1038/sj.bjp.0706429 PMC1760723

[B21] Tanaka K Gilroy S Jones AM Stacey G Extracellular ATP signaling in plants Trends Cell Biol 2010 20 601 608 10.1016/j.tcb.2010.07.005 20817461 PMC4864069

[B22] Clark G Roux SJ Extracellular nucleotides: Ancient signaling molecules Plant Sci 2009 177 239 244 10.1016/j.plantsci.2009.05.004

[B23] Roux SJ Steinebrunner I Extracellular ATP: an unexpected role as signaler in plants Trends Plant Sci 2007 12 522 527 10.1016/j.tplants.2007.09.003 17928260

[B24] Mouline K Very AA Gaymard F Boucherez J Pilot G Devic M Bouchez D Thibaud JB Sentenac H Pollen tube development and competitive ability are impaired by disruption of a Shaker K(+) channel in Arabidopsis Genes Dev 2002 16 339 350 10.1101/gad.213902 11825875 PMC155331

[B25] Nakagawa T Suzuki T Murata S Nakamura S Hino T Maeo K Tabata R Kawai T Tanaka K Niwa Y Improved Gateway binary vectors: high-performance vectors for creation of fusion constructs in transgenic analysis of plants Biosci Biotechnol Biochem 2007 71 2095 2100 10.1271/bbb.70216 17690442

[B26] Becker D Kemper E Schell J Masterson R New plant binary vectors with selectable markers located proximal to the left T-DNA border Plant Mol Biol 1992 20 1195 1197 10.1007/BF00028908 1463855

[B27] Clough SJ Bent AF Floral dip: a simplified method for Agrobacterium-mediated transformation of Arabidopsis thaliana Plant J 1998 16 735 743 10.1046/j.1365-313x.1998.00343.x 10069079

[B28] Sun Y Distribution and expression of apyrases in pea and Arabidopsis. PhD thesis 2003 The University of Texas at Austin: Botany Department

[B29] Haseloff J Siemering KR Prasher DC Hodge S Removal of a cryptic intron and subcellular localization of green fluorescent protein are required to mark transgenic Arabidopsis plants brightly Proc Natl Acad Sci U S A 1997 94 2122 2127 10.1073/pnas.94.6.2122 9122158 PMC20051

[B30] Payne CT Zhang F Lloyd AM GL3 encodes a bHLH protein that regulates trichome development in Arabidopsis through interaction with GL1 and TTG1 Genetics 2000 156 1349 1362 11063707 10.1093/genetics/156.3.1349 PMC1461316

[B31] Steinebrunner I Landschreiber M Krause-Buchholz U Teichmann J Rödel G HCC1, the Arabidopsis homologue of the yeast mitochondrial copper chaperone SCO1, is essential for embryonic development J Exp Bot 2011 62 319 330 10.1093/jxb/erq269 21041373

[B32] Geldner N Denervaud-Tendon V Hyman DL Mayer U Stierhof YD Chory J Rapid, combinatorial analysis of membrane compartments in intact plants with a multicolor marker set Plant J 2009 59 169 178 10.1111/j.1365-313X.2009.03851.x 19309456 PMC4854200

[B33] Seigneurin-Berny D Salvi D Dorne AJ Joyard J Rolland N Percoll-purified and photosynthetically active chloroplasts from Arabidopsis thaliana leaves Plant Physiol Biochem 2008 46 951 955 10.1016/j.plaphy.2008.06.009 18707896

[B34] Pernthaler A Pernthaler J Amann R Kowalchuk GA, Bruijn FJI, Head M, Akkermans ADL, Elsas JD Sensitive multi-color fluorescence in situ hybridization for the identification of environmental microorganisms Molecular Microbial Ecology Manual Volume 3.11 2004 Second Dordrecht: Kluwer Academic Publishers 711 726

[B35] Dettmer J Hong-Hermesdorf A Stierhof YD Schumacher K Vacuolar H+-ATPase activity is required for endocytic and secretory trafficking in Arabidopsis Plant Cell 2006 18 715 730 10.1105/tpc.105.037978 16461582 PMC1383645

[B36] Dent JA Polson AG Klymkowsky MW A whole-mount immunocytochemical analysis of the expression of the intermediate filament protein vimentin in Xenopus Development 1989 105 61 74 2806118 10.1242/dev.105.1.61

[B37] Kurth T Weiche S Vorkel D Kretschmar S Menge A Histology of plastic embedded amphibian embryos and larvae Genesis 2012 50 235 250 10.1002/dvg.20821 22083609

[B38] Zheng H Kunst L Hawes C Moore I A GFP-based assay reveals a role for RHD3 in transport between the endoplasmic reticulum and Golgi apparatus Plant J 2004 37 398 414 14731265 10.1046/j.1365-313x.2003.01969.x

[B39] Antifadehttp://www.riedlab.nci.nih.gov/protocols.asp

[B40] Schindelin J Arganda-Carreras I Frise E Kaynig V Longair M Pietzsch T Preibisch S Rueden C Saalfeld S Schmid B Fiji: an open-source platform for biological-image analysis Nat Methods 2012 9 676 682 10.1038/nmeth.2019 22743772 PMC3855844

[B41] Slot JW Geuze HJ Cryosectioning and immunolabeling Nat Protoc 2007 2 2480 2491 10.1038/nprot.2007.365 17947990

[B42] Tognoli L Marre E Purification and characterization of a divalent cation-activated ATP-ADPase from pea stem microsomes Biochim Biophys Acta 1981 642 1 14 10.1016/0005-2736(81)90132-2 6261809

[B43] Steinebrunner I Jeter C Song C Roux S Molecular and biochemical comparison of two different apyrases from Arabidopsis thaliana Plant Physiol Biochem 2000 38 913 922 10.1016/S0981-9428(00)01209-2

[B44] Keppler A Kindermann M Gendreizig S Pick H Vogel H Johnsson K Labeling of fusion proteins of O6-alkylguanine-DNA alkyltransferase with small molecules in vivo and in vitro Methods 2004 32 437 444 10.1016/j.ymeth.2003.10.007 15003606

[B45] Brecht A Gibbs T Self labeling protein tags Bioforum 2005 6 50 51

[B46] Knowles AF The GDA1_CD39 superfamily: NTPDases with diverse functions Purinergic Signal 2011 7 21 45 10.1007/s11302-010-9214-7 21484095 PMC3083126

[B47] Scott MS Calafell SJ Thomas DY Hallett MT Refining protein subcellular localization PLoS Comput Biol 2005 1 e66 10.1371/journal.pcbi.0010066 16322766 PMC1289393

[B48] Wilkinson J Twell D Lindsey K Activities of CaMV 35 S and nos promoters in pollen: implications for field release of transgenic plants J Exp Bot 1997 48 265 275 10.1093/jxb/48.2.265

[B49] McMurray MA Thorner J Septin stability and recycling during dynamic structural transitions in cell division and development Curr Biol 2008 18 1203 1208 10.1016/j.cub.2008.07.020 18701287 PMC2562167

[B50] Gautier A Juillerat A Heinis C Correa IR Jr Kindermann M Beaufils F Johnsson K An engineered protein tag for multiprotein labeling in living cells Chem Biol 2008 15 128 136 10.1016/j.chembiol.2008.01.007 18291317

[B51] Tomat E Nolan EM Jaworski J Lippard SJ Organelle-specific zinc detection using zinpyr-labeled fusion proteins in live cells J Am Chem Soc 2008 130 15776 15777 10.1021/ja806634e 18973293 PMC2645946

[B52] Provost CR Sun L Fluorescent labeling of COS-7 expressing SNAP-tag fusion proteins for live cell imaging J Vis Exp 2010 39 e1876 10.3791/1876 PMC3152857 20485262

[B53] van Gijlswijk RP Zijlmans HJ Wiegant J Bobrow MN Erickson TJ Adler KE Tanke HJ Raap AK Fluorochrome-labeled tyramides: use in immunocytochemistry and fluorescence in situ hybridization J Histochem Cytochem 1997 45 375 382 10.1177/002215549704500305 9071319

[B54] Takahashi H Toyoda K Hirakawa Y Morishita K Kato T Inagaki Y Ichinose Y Shiraishi T Localization and responsiveness of a cowpea apyrase VsNTPase1 to phytopathogenic microorganisms J Gen Plant Pathol 2006 72 143 151 10.1007/s10327-005-0267-3

[B55] Llopis J McCaffery JM Miyawaki A Farquhar MG Tsien RY Measurement of cytosolic, mitochondrial, and Golgi pH in single living cells with green fluorescent proteins Proc Natl Acad Sci U S A 1998 95 6803 6808 10.1073/pnas.95.12.6803 9618493 PMC22642

[B56] Langhans M Hawes C Hillmer S Hummel E Robinson DG Golgi regeneration after Brefeldin A treatment in BY-2 cells entails stack enlargement and cisternal growth followed by division Plant Physiol 2007 145 527 538 10.1104/pp.107.104919 17704232 PMC2048719

[B57] Nelson BK Cai X Nebenfuhr A A multicolored set of in vivo organelle markers for co-localization studies in Arabidopsis and other plants Plant J 2007 51 1126 1136 10.1111/j.1365-313X.2007.03212.x 17666025

[B58] Lam SK Cai Y Tse YC Wang J Law AH Pimpl P Chan HY Xia J Jiang L BFA-induced compartments from the Golgi apparatus and trans-Golgi network/early endosome are distinct in plant cells Plant J 2009 60 865 881 10.1111/j.1365-313X.2009.04007.x 19709389

[B59] Zheng H Camacho L Wee E Batoko H Legen J Leaver CJ Malho R Hussey PJ Moore I A Rab-E GTPase mutant acts downstream of the Rab-D subclass in biosynthetic membrane traffic to the plasma membrane in tobacco leaf epidermis Plant Cell 2005 17 2020 2036 10.1105/tpc.105.031112 15972698 PMC1167549

[B60] Speth EB Imboden L Hauck P He SY Subcellular localization and functional analysis of the Arabidopsis GTPase RabE Plant Physiol 2009 149 1824 1837 10.1104/pp.108.132092 19233904 PMC2663744

[B61] Wee EG Sherrier DJ Prime TA Dupree P Targeting of active sialyltransferase to the plant Golgi apparatus Plant Cell 1998 10 1759 1768 9761801 10.1105/tpc.10.10.1759 PMC143948

[B62] Camacho L Smertenko AP Perez-Gomez J Hussey PJ Moore I Arabidopsis Rab-E GTPases exhibit a novel interaction with a plasma-membrane phosphatidylinositol-4-phosphate 5-kinase J Cell Sci 2009 122 4383 4392 10.1242/jcs.053488 19903693

[B63] Uemura T Ueda T Ohniwa RL Nakano A Takeyasu K Sato MH Systematic analysis of SNARE molecules in Arabidopsis: dissection of the post-Golgi network in plant cells Cell Struct Funct 2004 29 49 65 10.1247/csf.29.49 15342965

[B64] Conchon S Cao X Barlowe C Pelham HR Got1p and Sft2p: membrane proteins involved in traffic to the Golgi complex EMBO J 1999 18 3934 3946 10.1093/emboj/18.14.3934 10406798 PMC1171469

[B65] Bolte S Cordelieres FP A guided tour into subcellular colocalization analysis in light microscopy J Microsc 2006 224 213 232 10.1111/j.1365-2818.2006.01706.x 17210054

[B66] Mitsui T Honma M Kondo T Hashimoto N Kimura S Igaue I Structure and function of the Golgi complex in rice cells (II. Purification and characterization of Golgi membrane-bound nucleoside diphosphatase) Plant Physiol 1994 106 119 125 12232309 10.1104/pp.106.1.119 PMC159506

[B67] Mikami S Suganuma R Hori H Mitsui T Purification and characterization of Golgi membrane-bound nucleoside diphosphatase from suspension-cultured cells of sycamore (Acer pseudoplatanus L.) Plant Biotechnol 2001 18 259 265 10.5511/plantbiotechnology.18.259

[B68] Monshausen GB Bibikova TN Weisenseel MH Gilroy S Ca2+ regulates reactive oxygen species production and pH during mechanosensing in Arabidopsis roots Plant Cell 2009 21 2341 2356 10.1105/tpc.109.068395 19654264 PMC2751959

[B69] Krogh A Larsson B von Heijne G Sonnhammer EL Predicting transmembrane protein topology with a hidden Markov model: application to complete genomes J Mol Biol 2001 305 567 580 10.1006/jmbi.2000.4315 11152613

[B70] Nicol F His I Jauneau A Vernhettes S Canut H Hofte H A plasma membrane-bound putative endo-1,4-beta-D-glucanase is required for normal wall assembly and cell elongation in Arabidopsis EMBO J 1998 17 5563 5576 10.1093/emboj/17.19.5563 9755157 PMC1170885

[B71] Zeng Keegstra K AtCSLD2 is an integral Golgi membrane protein with its N-terminus facing the cytosol Planta 2008 228 823 838 10.1007/s00425-008-0785-2 18642024

[B72] Hua S Sun Z Support vector machine approach for protein subcellular localization prediction Bioinformatics 2001 17 721 728 10.1093/bioinformatics/17.8.721 11524373

[B73] Emanuelsson O Nielsen H Brunak S von Heijne G Predicting subcellular localization of proteins based on their N-terminal amino acid sequence J Mol Biol 2000 300 1005 1016 10.1006/jmbi.2000.3903 10891285

[B74] Horton P Park KJ Obayashi T Fujita N Harada H Adams-Collier CJ Nakai K WoLF PSORT: protein localization predictor Nucleic Acids Res 2007 35 W585 W587 10.1093/nar/gkm259 17517783 PMC1933216

[B75] Small I Peeters N Legeai F Lurin C Predotar: A tool for rapidly screening proteomes for N-terminal targeting sequences Proteomics 2004 4 1581 1590 10.1002/pmic.200300776 15174128

[B76] PSORT Predictionhttp://psort.hgc.jp/form.html

[B77] Clark G Fraley D Steinebrunner I Cervantes A Onyirimba J Liu A Torres J Tang W Kim J Roux SJ Extracellular nucleotides and apyrases regulate stomatal aperture in Arabidopsis Plant Physiol 2011 156 1740 1753 10.1104/pp.111.174466 21636723 PMC3149927

[B78] Kim SH Yang SH Kim TJ Han JS Suh JW Hypertonic stress increased extracellular ATP levels and the expression of stress-responsive genes in Arabidopsis thaliana seedlings Biosci Biotechnol Biochem 2009 73 1252 1256 10.1271/bbb.80660 19502745

[B79] Song C Steinebrunner I Wang X Stout SC Roux SJ Extracellular ATP induces the accumulation of superoxide via NADPH oxidases in Arabidopsis thaliana Plant Physiol 2006 140 1222 1232 10.1104/pp.105.073072 16428598 PMC1435826

[B80] Dunkley TP Hester S Shadforth IP Runions J Weimar T Hanton SL Griffin JL Bessant C Brandizzi F Hawes C Mapping the Arabidopsis organelle proteome Proc Natl Acad Sci U S A 2006 103 6518 6523 10.1073/pnas.0506958103 16618929 PMC1458916

[B81] Parsons HT Christiansen K Knierim B Carroll A Ito J Batth TS Smith-Moritz AM Morrison S McInerney P Hadi MZ Isolation and proteomic characterization of the Arabidopsis Golgi defines functional and novel components involved in plant cell wall biosynthesis Plant Physiol 2012 159 12 26 10.1104/pp.111.193151 22430844 PMC3375956

[B82] Orellana A Neckelmann G Norambuena L Topography and function of Golgi uridine-5’-diphosphatase from pea stems Plant Physiol 1997 114 99 107 12223692 10.1104/pp.114.1.99 PMC158283

[B83] Staver MJ Glick K Baisted DJ Uridine diphosphate glucose-sterol glucosyltransferase and nucleoside diphosphatase activities in etiolated pea seedlings Biochem J 1978 169 297 303 204295 10.1042/bj1690297 PMC1184167

[B84] Perrin R Wilkerson C Keegstra K Golgi enzymes that synthesize plant cell wall polysaccharides: finding and evaluating candidates in the genomic era Plant Mol Biol 2001 47 115 130 10.1023/A:1010675213873 11554467

[B85] Neckelmann G Orellana A Metabolism of uridine 5’-diphosphate-glucose in Golgi vesicles from pea stems Plant Physiol 1998 117 1007 1014 10.1104/pp.117.3.1007 9662543 PMC34916

[B86] Abeijon C Yanagisawa K Mandon EC Hausler A Moremen K Hirschberg CB Robbins PW Guanosine diphosphatase is required for protein and sphingolipid glycosylation in the Golgi lumen of Saccharomyces cerevisiae J Cell Biol 1993 122 307 323 10.1083/jcb.122.2.307 8391537 PMC2119650

[B87] D’Alessio C Caramelo JJ Parodi AJ Absence of nucleoside diphosphatase activities in the yeast secretory pathway does not abolish nucleotide sugar-dependent protein glycosylation J Biol Chem 2005 280 40417 40427 10.1074/jbc.M503149200 16172132

[B88] Hummel E Osterrieder A Robinson DG Hawes C Inhibition of Golgi function causes plastid starch accumulation J Exp Bot 2010 61 2603 2614 10.1093/jxb/erq091 20423939 PMC2882258

[B89] Nagy B Jencks WP Depolymerization of F-Actin by concentrated solutions of salt and denaturing agents J Am Chem Soc 1965 87 2480 2488 10.1021/ja01089a030 14327156

[B90] Cheitlin RA Ramachandran J Purification of rat adrenocortical actin and its use in an immunoprecipitation assay to quantitate cellular actin Biochim Biophys Acta 1986 883 383 387 10.1016/0304-4165(86)90332-6 3527278

