# Supplementary material for: The Arabidopsis apyrase AtAPY1 is localized in the Golgi instead of the extracellular space
Source: BMC Plant Biol. 2012 Jul 31;12:123. doi: 10.1186/1471-2229-12-123 (PMC3511161; doi:10.1186/1471-2229-12-123)
Supplement: Additional file 1 — AtAPY1-SNAP DNA sequence. The AtAPY1-SNAP DNA sequence present in the AtAPY1::AtAPY1-SNAP transgenic lines is shown. [file 1471-2229-12-123-S1.pdf]

ATGACGGCGAAGCGAGCGATCGGACGGCACGAATCCCTTGCTGACAAGGTCCAT
CGACATCGTGGTCTTCTACTTGTGATTTCGATCCCCATTGTGTTGATAGCTCTTGT
GCTTCTGTTAATGCCGGGGACGTCGACGTCCGTCTCTGTCATCGAGTACACGATG
AAAAACCACGAGGGAGGTTCCAATTCGAGGGGTCCGAAGAATTACGCTGTGATT
TTTGATGCTGGAAGTTCTGGAAGCCGTGTGCATGTTTACTGTTTCGATCAGAATTT
GGATCTTGTTCCTTTGGAGAATGAGCTCGAGCTCTTCTTACAGCTAAAACCGGGTT
TAAGTGCATATCCTAATGATCCTCGGCAATCAGCAAACTCTTTAGTAACTCTTCTG
GACAAAGCAGAAGCTTCCGTTCCCCGTGAGTTGCGTCCAAAGACTCCTGTCAGAG
TTGGGGCAACTGCAGGTTTGAGAGCTTTGGGTCACCAAGCCTCTGAAAACATTTT
GCAAGCGGTTAGGGAGCTCCTCAAAGGTAGAAGTAGGCTGAAGACTGAGGCAAA
TGCAGTGACTGTTCTGGATGGTACTCAGGAAGGATCTTATCAGTGGGTGACAATT
AATTACTTGCTAAGGACTTTGGGAAAGCCGTACTCGGACACAGTTGGAGTGGTTG
ATCTTGGAGGGGGGTCGGTTCAAATGGCATATGCTATACCAGAGGAAGATGCGG
CAACTGCACCAAAACCAGTAGAAGGCGAGGATTCTTATGTCAGAGAAATGTATTT
GAAGGGACGAAAGTATTTCCTCTATGTTCATAGCTACCTACATTACGGGTTACTG
GCTGCTCGGGCTGAGATTTTGAAAGTTTCTGAGGACTCTAACAACCCCTGTATCG
CGACTGGATATGCTGGTACCTACAAATATGGAGGAAAAGCGTTTAAAGCTGCAG
CTTCTCCATCCGGTGCAAGTCTAGATGAGTGCCGGCGAGTAGCTATTAACGCACT
CAAAGTCAATAATTCATTGTGTACACACATGAAATGCACTTTTGGTGGAGTATGG
AATGGTGGAGGCGGTGGTGGCCAGAAGAAAATGTTTGTTGCATCATTTTTCTTCG
ATCGAGCCGCAGAGGCTGGTTTTGTTGACCCAAACCAACCTGTGGCTGAGGTTCG
ACCACTTGACTTTGAGAAAGCGGCCAACAAAGCTTGTAACATGAGAATGGAAGA
AGGGAAATCGAAGTTCCCACGTGTGGAGGAAGATAATCTTCCTTACTTGTGCTTG
GATCTTGTTTACCAATATACACTTCTCGTCGATGGATTCGGATTGAAGCCATCACA
GACAATAACGTTAGTGAAGAAGGTGAAATACGGAGATTACGCCGTGGAAGCTGC
GTGGCCACTAGGAAGCGCAATAGAAGCAGTATCCTCACCACCTTATCTTGACGCA
TGCGAGCTCGGTACCCCGGGTCGACGGGGCAATAGTAAAGGAGAAGAACTTTTC
ACTGGAGTTGTCCCAATTCTTGTTGAATTAGATGGTGATGTTAATGGGCACAAAT
TTTCTGTCAGTGGAGAGGGTGAAGGTGATGCAACATACGGAAAACTTACCCTTAA
ATTTATTTGCACTACTGGAAAACTACCTGTTCCATGGCCAACACTTGTCACTACTT
TCTCTTATGGTGTTCAATGCTTTTCAAGATACCCAGATCATATGAAGCGGCACGA
CTTCTTCAAGAGCGCCATGCCTGAGGGATACGTGCAGGAGAGGACCATCTTCTTC
AAGGACGACGGGAACTACAAGACACGTGCTGAAGTCAAGTTTGAGGGAGACACC
CTCGTCAACAGGATCGAGCTTAAGGGAATCGATTTCAAGGAGGACGGAAACATC
CTCGGCCACAAGTTGGAATACAACTACAACTCCCACAACGTATACATCATGGCCG
ACAAGCAAAAGAACGGCATCAAAGCCAACTTCAAGACCCGCCACAACATCGAAG
ACGGCGGCGTGCAACTCGCTGATCATTATCAACAAAATACTCCAATTGGCGATGG
CCCTGTCCTTTTACCAGACAACCATTACCTGTCCACACAATCTGCCCTTTCGAAAG
ATCCCAACGAAAAGAGAGACCACATGGTCCTTCTTGAGTTTGTAACAGCTGCTGG
GATTACACATGGCATGGATGAACTATACAAATAA
