# Supplementary figures and images for: The Arabidopsis apyrase AtAPY1 is localized in the Golgi instead of the extracellular space
Source: BMC Plant Biol. 2012 Jul 31;12:123. doi: 10.1186/1471-2229-12-123 (PMC3511161; doi:10.1186/1471-2229-12-123)

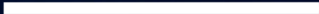

Supplement: Additional file 3 — Immunofluorescence of pre-imbedding labeled AtAPY1-GFP in root hair. Roots from AtAPY1-GFP expressing seedlings were immunostained whole mount, embedded in resin and sectioned. The sections were successively incubated with α-GFP and secondary α-rabbit Fab fragments coupled with Alexa Fluor 488. A 3-μm cross-section of a root hair is shown. The Alexa Fluor 488 fluorescence is shown in green. Scale bar equals 20 μm. [file 1471-2229-12-123-S3.pdf]

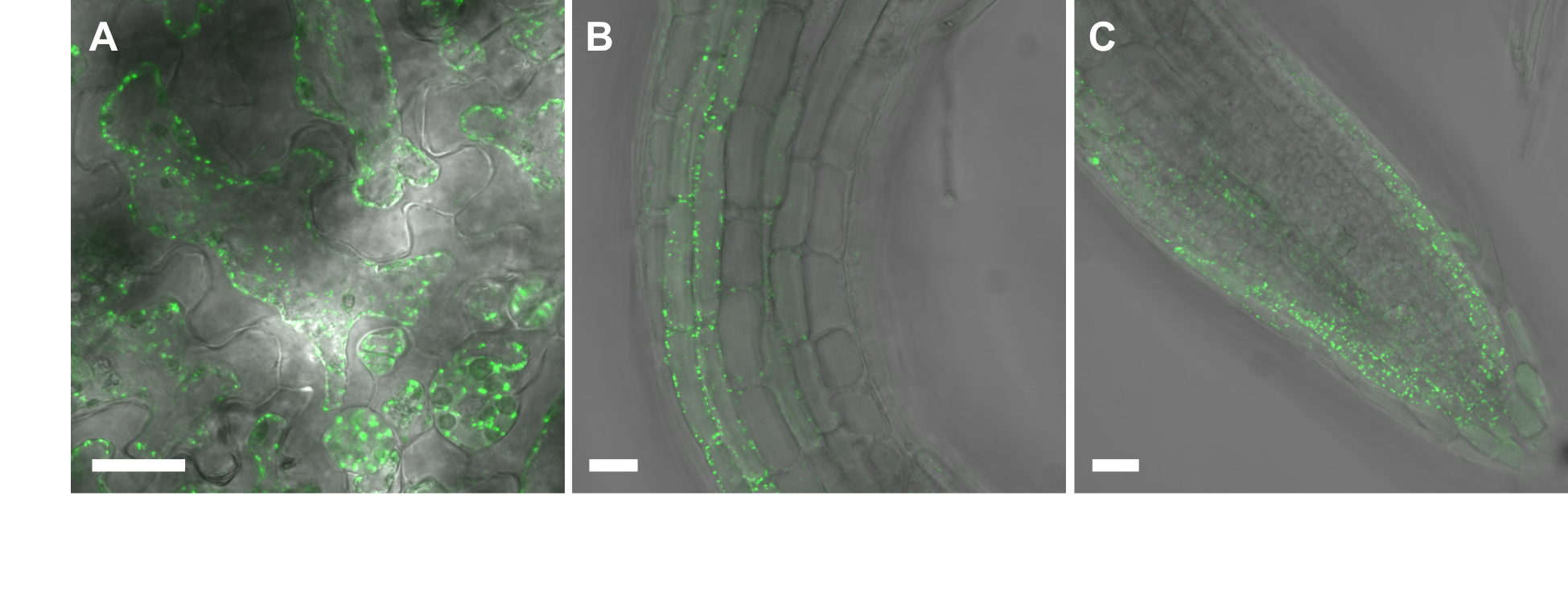

Supplement: Additional file 4 — Live imaging of AtAPY1-GFP in various cell types. CLSM images of various tissues in AtAPY1-GFP expressing seedlings show the GFP signals in green overlaid with bright field view. (A) Cotyledon epidermis, (B) hypocotyl and (C) root tip. Scale bars = 20 μm. [file 1471-2229-12-123-S4.tiff]

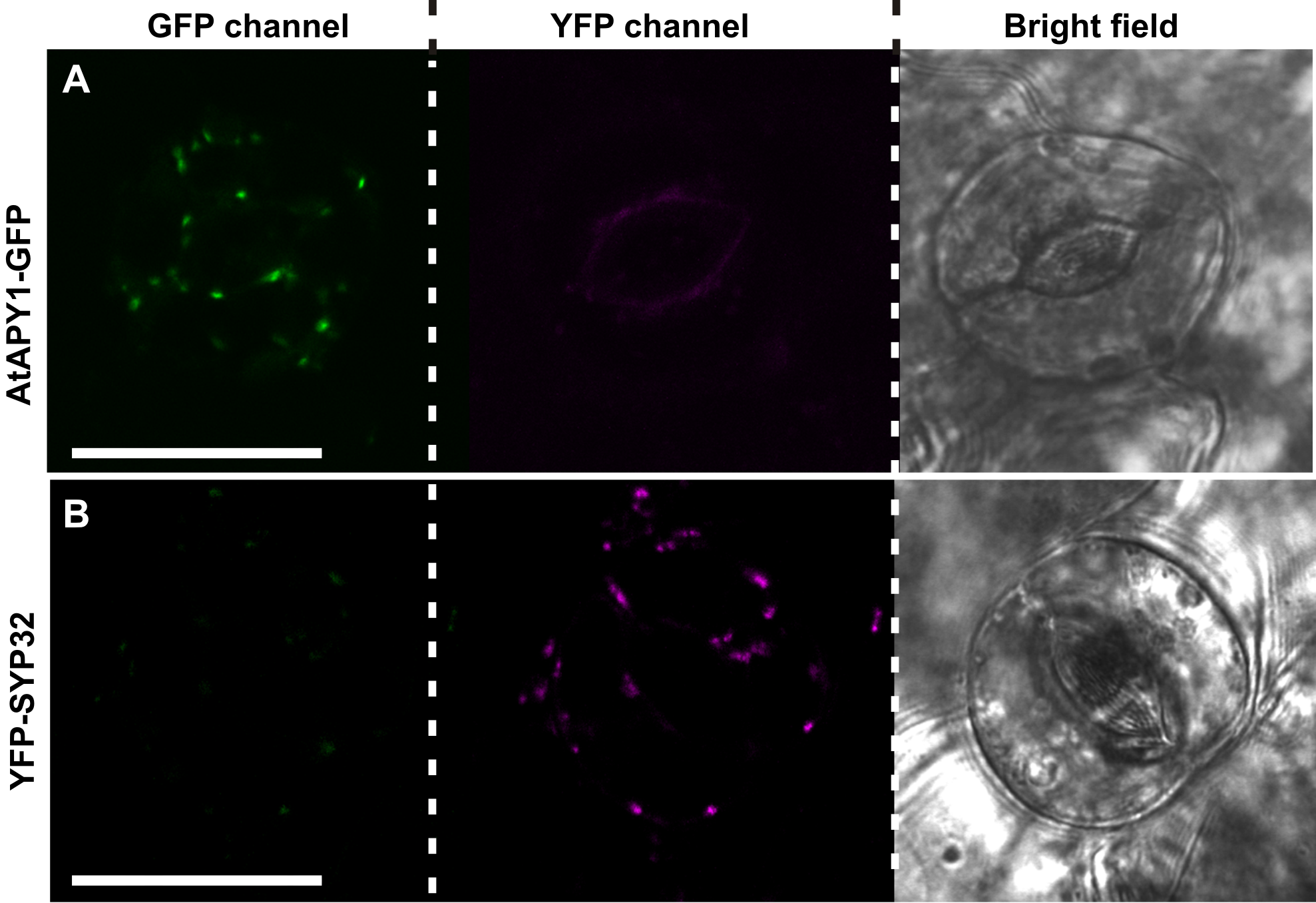

Supplement: Additional file 5 — Specificity of the imaging settings for the detection of GFP and YFP fluorescence. The epidermis of cotyledons from transgenic plant lines was imaged with the GFP and YFP settings outlined under Methods for the “CLSM”. The GFP and YFP fluorescence is shown in green and magenta, respectively. Scale bars = 20 μm. (A) The identical epidermal section with two guard cells from plants expressing AtAPY1-GFP only was imaged sequentially with the GFP and YFP settings. Dot-like signals appeared in the GFP detection channel only. Only weak autofluorescence of the thickened cell wall around the stomate was visible in the YFP detection channel. (B) The identical epidermal section with two guard cells from plants expressing YFP-SYP32 only was imaged sequentially with the GFP and YFP settings. Here, only very weak signals were detectable in the GFP detection channel, but strong YFP fluorescence appeared with the YFP-specific excitation and detection. [file 1471-2229-12-123-S5.tiff]

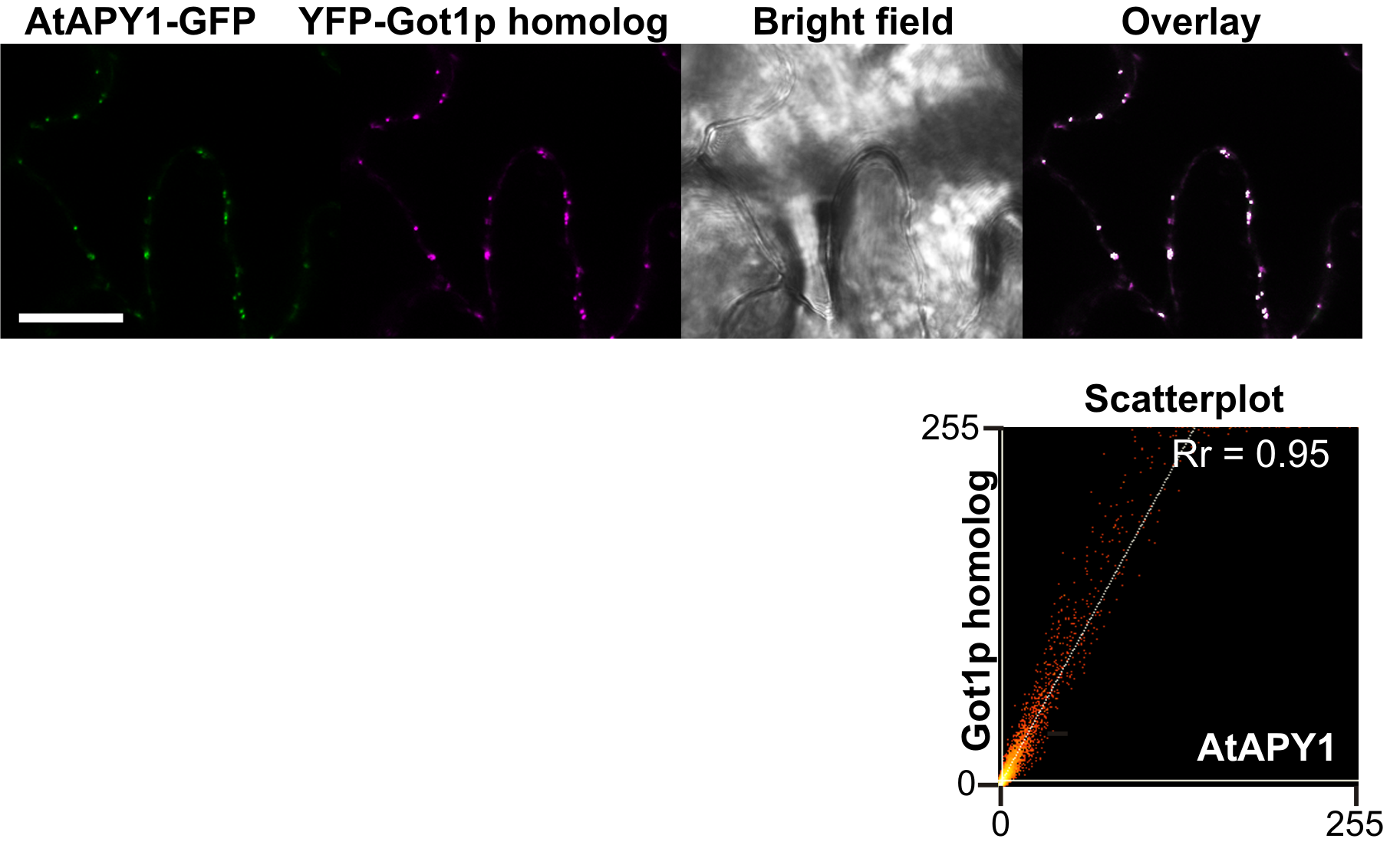

Supplement: Additional file 6 — Co-localization analysis of AtAPY1-GFP and YFP-Got1p homolog. CLSM images of epidermal cells of cotyledons from transgenic lines co-expressing AtAPY1-GFP and YFP-Got1 phomolog were taken. The GFP fluorescence is shown in green and the YFP fluorescence in magenta. The bright field-type image was acquired with the transmitted light detector. The fluorescence signals for AtAPY1-GFP and YFP-Got1p homolog were detected separately and merged for co-localization with the “Co-localization Finder” plugin of ImageJ. Co-localization of the two proteins is depicted as white signals. The corresponding scatterplot was analyzed with the ImageJ “Colocalization Threshold” and “Coloc2” tool from ImageJ. The x-axis represents the pixel intensities from the GFP channel and the y-axis from the YFP channel. Rr = Pearson’s correlation coefficient. Scale bar = 20 μm. [file 1471-2229-12-123-S6.tiff]

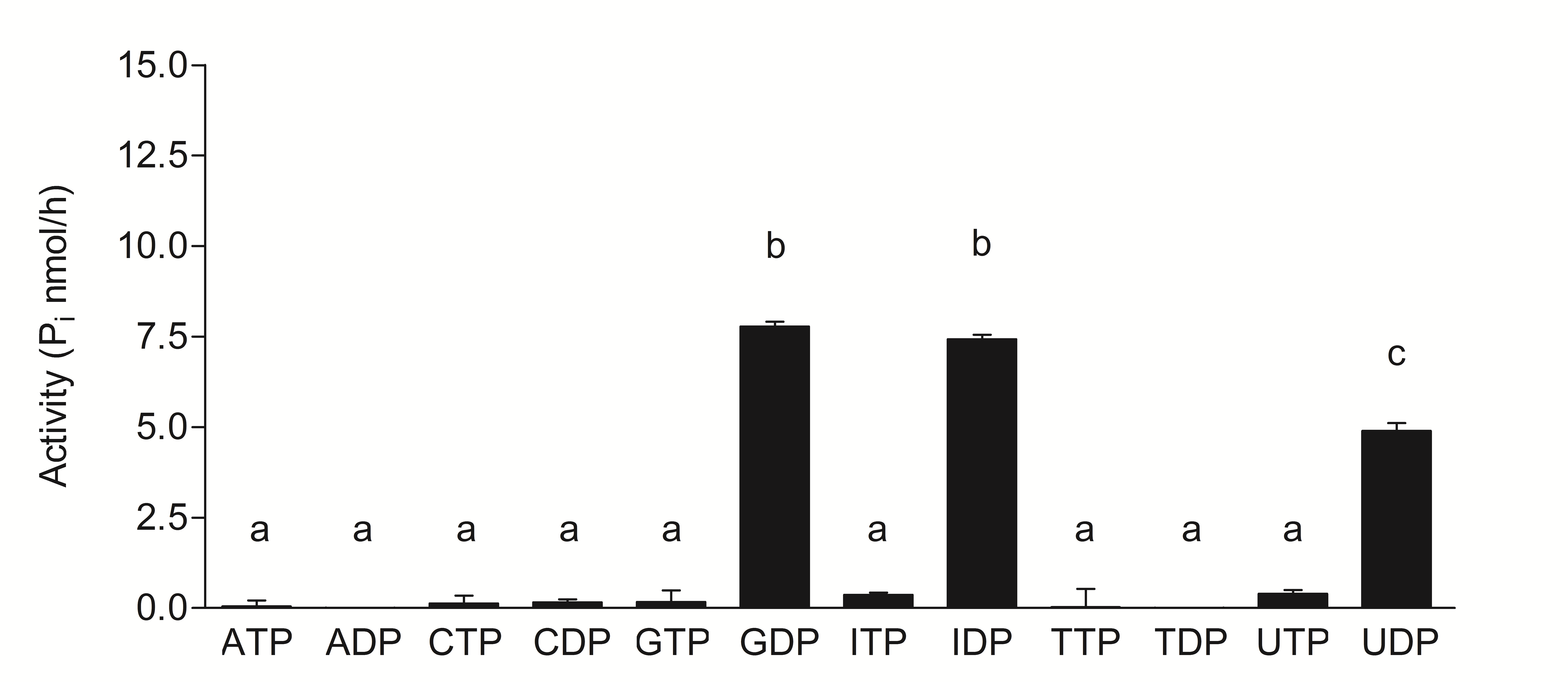

Supplement: Additional file 7 — Substrate specificity of AtAPY1-GFP at pH 5.5. The activity of AtAPY1-GFP at pH 5.5 in the presence of various substrates was measured. No activity was detectable with AMP as substrate (data not shown). Different letters above the columns indicate mean values that are significantly different from one other (p<0.05, Tukey test). Error bars represent standard deviations of two phosphate measurements from one reaction (see Methods). Abbreviation: Pi, inorganic phosphate. [file 1471-2229-12-123-S7.png]

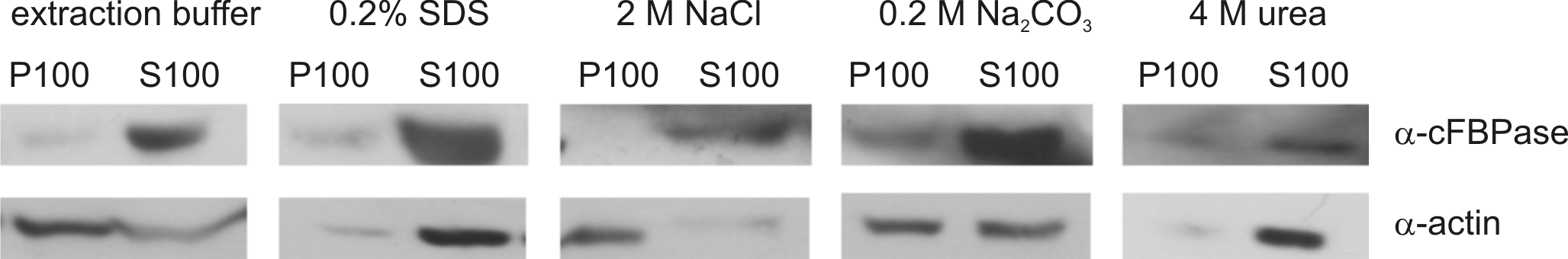

Supplement: Additional file 8 — Analysis of the purity of microsomal membrane and soluble protein fractions. Protein extracts from transgenic plants expressing 35S::AtAPY1-GFP were treated with either extraction buffer, 0.2% SDS, 2 M NaCl, 0.2 M Na2CO3 or 4 M urea and then centrifuged at 100,000 g to obtain microsomal membrane fractions (P100) and supernatants (S100). Proteins from each fraction (40 μg each) were subjected to Western blot analysis. The enrichment of microsomal and insoluble proteins in the P100 fractions and of soluble proteins in the S100 fractions was confirmed with antibodies against marker proteins. The 37-kDa cytosolic fructose-1,6-bisphosphatase (cFBPase) served as a marker protein for soluble proteins. Actin (45 kDa) was used as a marker protein for insoluble proteins under actin polymerizing conditions found in the extraction buffer and 2 M NaCl [89] and as a soluble marker under actin depolymerizing conditions such as 0.2 M Na2CO3, 0.2% SDS and 4 M urea [89,90]. [file 1471-2229-12-123-S8.tiff]
